# Supplementary material for: miR‐200/375 control epithelial plasticity‐associated alternative splicing by repressing the RNA‐binding protein Quaking
Source: EMBO J. 2018 Jun 6;37(13):e99016. doi: 10.15252/embj.201899016 (PMC6028027; doi:10.15252/embj.201899016)
Supplement: Supplementary file 1 — Appendix [file EMBJ-37-e99016-s001.pdf]

Appendix table of contents

Appendix Supplementary Methods

Appendix Supplementary Methods Figure S1 – QKI-5 HITS-CLIP

Appendix Figure S1. Gene ontology analysis of QKI-5 regulated spliced genes during EMT

Appendix Figure S2. Expression of splicing factors in response to miR-200c transfection

APPENDIX SUPPLEMENTARY METHODS

QKI-5-CLIP and sequencing

A summary of the methodology used is shown in Appendix Supplementary Methods Figure S1A

UV crosslinking and preparation of lysates:

MesHMLE cells were grown in 100mm plates to ~90% confluency, rinsed once with ice-cold PBS, and irradiated with 600 mJ/cm2 in ice-cold PBS using a UV Stratalinker-1800 (Agilent). Cells were collected by scraping, washed in PBS, and stored at -80°C as one pellet per plate. Each pellet was resuspended using 200 µl of 1 X QLB (1 X PBS, 0.3% SDS, 0.5% deoxycholate, 0.5% Igepal, EDTA-free Complete protease inhibitor cocktail (PIC; Roche, 11873580001) for 15 min on ice to liberate QKI from high molecular weight complexes, followed by addition of 400 µl of 1 X QDB (1 X PBS, 0.5% deoxycholate, 0.5% Igepal, PIC) and trituration by passing through a 21G needle and syringe 5 times. DNA was digested with 20 µl RQ1 DNAse (Promega, M6101) at 37°C for 10 min on a Thermomixer (750 rpm, Eppendorf). RNA was partially digested with RNase 1 (ThermoFisher, AM2295) by adding 6 µl of 1:50 diluted RNase 1 in 1 X PBS at 37°C for 5 min on a Thermomixer (750 rpm), then returned to ice. Lysates were centrifuged at 21,000 x g for 20 min at 4°C and supernatant transferred to a fresh tube.

Immunoprecipitation:

QKI-RNA complexes were immunoprecipitated using a QKI5 specific antibody (Bethyl, A300-183A) with a rabbit IgG antibody (Santa Cruz sc-2027) used as a control (Appendix Supplementary Methods Figure S1B,C). Antibodies (5 µg) were conjugated to 100 µl protein A Dynabeads (ThermoFisher, 10002D) in PBS-Tw (1 X PBS, 0.05% Tween-20) for 45 min and washed three times with 1 X PXL (1 X PBS, 0.1% SDS, 0.5% sodium deoxycholate, 0.5% Igepal) before resuspending the beads with 500 µl of prepared lysate and rotating for 2 hr at 4°C. A further 2% of IP input lysate was set aside to be used as a size-matched input (SMin) as in van Nostrand et al 2016 (Van Nostrand *et al.*, 2016). Bound QKI-5-RNA complexes were washed twice each consecutively with ice cold 1 X PXL, 5 X PXL (5 X PBS, 0.1% SDS, 0.5% sodium deoxycholate, 0.5% Igepal), and 1 X PNK (50 mM Tris-Cl pH 7.5, 10 mM $MgCl_2$, and 0.5% Igepal).

3' end dephosphorylation and 3' linker ligation

Beads were first treated with T4 PNK (NEB, M0201L; 20 U in 80 µl reaction volume) in the absence of ATP at 37°C, 850 rpm for 20 min, to dephosphorylate 3' RNA ends followed by washes with 1 X PNK, 5 X PXL, and two washes with 1 X PNK at 4°C. The 3' preadenylated linker (NEBNext 3'SR adaptor for Illumina) was ligated to the RNA fragments on bead using RNA ligase I (NEB M0437M; 75 U in a 30 µl reaction volume, 15% PEG8000, 2.5% DMSO, 0.25 µM adaptor) in the absence of ATP at 22°C, 75 min with periodic mixing. Beads were washed with 1 X PNK, 1 X PNK + EGTA (50 mM Tris-Cl pH 7.5, 20 mM EGTA, and 0.5% Igepal), 5 X PXL, and two washes with 1X PNK at 4°C. Twenty percent of beads were removed and labelled with P^{32} γ-ATP using T4 PNK (according to Sutandy *et al* 2016 (Sutandy *et al.*, 2016)), washed then recombined with the unlabelled fraction to track the RNA in subsequent steps.

SDS PAGE, nitrocellulose transfer, and RNA extraction and size purification

QKI-5-RNA complexes were eluted with 40 µl 1 X Bolt LDS sample buffer (ThermoFisher) without reducing agent at 70°C for 10 min on a Thermomixer (1200 rpm). Samples were separated through Bolt 10% Bis-tris Plus gels (ThermoFisher) using Bolt MOPS SDS running buffer at 165 V for 47 min. Complexes were then transferred to nitrocellulose (Schleicher&Schuell, BA-85) by wet transfer using 1 X Bolt transfer buffer with 10% methanol (Appendix Supplementary Methods Figure S1D).Filters were placed on a phosphor screen and exposed using a Typhoon imager (GE). Nitrocellulose was cut as marked in Appendix Supplementary Methods Figure S1E and the RNA extracted by proteinase K digestion (2 mg/mL proteinase K, 100 mM Tris-HCl pH 7.5, 50 mM NaCl, 10 mM EDTA, 0.2% SDS) at 50°C for 60 min on a Thermomixer (1200 rpm) followed by extraction with acid phenol (ThermoFisher, AM9712) and precipitation with 1:1 isopropanol:ethanol. RNA was pelleted by centrifugation then separated on a 15% denaturing polyacrylamide gel (1:19 acrylamide, 1 X TBE, 7 M urea). The wet gel was wrapped in plastic wrap and exposed to a phosphor screen and imaged using a Typhoon. Gel slices were cut as marked in Appendix Supplementary Methods Figure S1F and the RNA eluted by the "crush and soak" method as previously described (Jensen & Darnell, 2008). Size match input controls (SMin) were prepared essentially as preciously described (Van Nostrand *et al.*, 2016) with the addition of RNA size selection alongside the QKI-associated RNA fragments.

Library Preparation

Reverse transcription, 5' linker ligation and amplification were performed essentially as previously described (Van Nostrand *et al.*, 2016) but using a custom synthesized 5' linker (IDT, 5'SRdeg /5Phos/NN NNN NNN NNG ATC GTC GGA CTG TAG AAC TCT GAA C/3SpC3/), and SR-RT primer for reverse transcription (IDT, AGACGTGTGCTCTTCCGATCT). Products were amplified for 17 (CLIP) or 9 (SMin) cycles using a common forward primer (NEBNext SR primer for Illumina) and barcoded reverse primers for each sample (NEBNext Index primers for Illumina). PCR products were purified using Qiagen Qiaquick PCR purification kit, separated on a 10% acrylamide (29:1) TBE non-denaturing gel, stained with SYBR Gold nucleic acid gel stain (ThermoFisher) and imaged on a ChemiDoc (BioRad). Products corresponding to an insert size of ~30 – 70 nt were excised from the gel as shown in Appendix Supplementary Methods Figure S1G and extracted by the "crush and soak" method as previously described (Jensen & Darnell, 2008). Library quality and quantity was assessed by Bioanalyzer (Agilent), Qubit (ThermoFisher) and qPCR, pooled and sequenced on an Illumina NextSeq 500 (1 x 75bp).

Processing and analysis of RNA-seq data

Sequencing read QC and mapping

Raw reads were adapter-trimmed and filtered for short sequences using cutadapt v1.3(Martin, 2011), in paired-end mode (parameters: minimum read length: 18 nt, maximum error-rate: 0.2, minimum adapter overlap: 5 nt). The resulting FASTQ files were analyzed and quality checked using FastQC. Reads were mapped against the human reference genome (build GRChr37/hg19) using the iGenomes UCSC hg19 gene annotations with TopHat2 v2.0.10 (Kim *et al.*, 2013) using mate inner distance and standard deviation values estimated from a random subsample of 500,000 reads (optional parameters: -p 4 --GTF <gene annotation gtf file> --library-type=fr-firststrand --mate-inner-dist=<mate inner distance> --mate-std-dev=<mate standard deviation>).

Differential gene expression analysis

Gene expression quantification and differential gene expression analysis was performed using cuffdiff command from cufflinks v2.1.1, providing a mask file containing

gene annotation for tRNAs, rRNAs and mitochondrial genes (parameters: --num-threads 8 --min-reps-for-js-test 2 --frag-bias-correct <genome.fa> --multi-read-correct --mask-file <mask.gtf><UCSC gene annotation.gtf>) (Trapnell *et al.*, 2013). Differential splicing (discussed below) was analysed concurrently.

Differential alternative splicing analysis

Differential splicing analysis is complex and different approaches often yield different findings (Liu *et al.*, 2014). To ensure our findings were robust, we assessed differential splicing using three diverse algorithms: rMATS (Shen *et al.*, 2014), DEXSeq (Anders *et al.*, 2012) and cuffdiff. DEXSeq was used according to the author's instructions (Anders *et al.*, 2012). Gene annotation was prepared by discarding non-standard human chromosomes and mitochondrial genes and 'flattening' the gtf using the provided script, dexseq_prepare_annotation.py. BAM files were sorted by read name and the reads in each genomic 'bin' were counted using the provided script, dexseq_count.py (parameters -p yes -s reverse -a 10 -f bam -r name). DEXSeq was run using a basic model (full model: ~ sample + exon + condition:exon; reduced model: ~ sample + exon). rMATS v3.0.9 (parameters: -gtf <UCSC gene annotation.gtf> -t paired -len 101 -analysis U) and the Cufflinks subprogram, cuffdiff (described above) were also used. As expected, we observed many differences for individual genes, however, globally, the findings based on any of the three algorithms were largely consistent, particularly for high confidence splicing changes. The figures presented in this work were produced using rMATS results because of the useful classification of splicing event types. However, to compare our findings to QKI-correlated splicing in TCGA samples and to choose genes for lab-validation, the results from all three algorithms were combined to produce a single splicing metric (provided in Table EV6) as follows. Firstly, for each algorithm separately, we ranked all genes according to the evidence for differential splicing. For cuffdiff, genes were ranked by the minimum p-value of any isoform for that gene. For rMATS, the top-ranked genes were required to pass cutoffs for FDR (FDR $\leq$ 0.05) and change in PSI (ΔPSI $\geq$ 10%), and were then ordered by FDR. Subsequent ranks were given to genes which passed the change in PSI but not the FDR cutoff, ordered by p-value; then genes which passed the p-value cutoff but not PSI cutoff, ordered by p-value; and finally, all remaining genes, ordered by p-value. For DEXSeq, genes were ranked by their gene-level p-value. To combine the ranks from the three methods, firstly, an upper limit (poorest ranking) of 3000 was applied to the rankings and the squares of the ranks were calculated. Next, to reduce the impact of minor inconsistencies between algorithms, for each gene, the value of the largest (poorest) of the three ranks was halved. Finally, the overall

ranking metric was then calculated as the square root of the sum of the resulting three values. For comparison to TCGA QKI-correlated splicing, genes were ordered by summing the ranks from the EMT and QKI knockdown experiments and filtering for direct targets of QKI in QKI HITS-CLIP.

HITS-CLIP analysis of endogenous QKI-5

QC, adapter trimming and read mapping was performed as described for RNA-seq but for single-end reads. Next, the molecular barcodes of reads which had both a) 5' ends mapping to the same position and b) identical alignment CIGAR strings, were analysed using Python code and reads with barcodes sequences ≤1 edit distance apart were classified as duplicates and collapsed to a single read. Low-quality alignments were discarded using samtools(Li *et al.*, 2009) (view parameter: -bq 10) and alignments from samples prepared using the same antibody and biological input but with other technical variations in library preparation method were pooled prior to peak calling.

Peak calling was performed separately for each strand using MACS2 2.1.1.20160309 (Zhang *et al.*, 2008) (callpeak program parameters: -t <experimental bam file> -c <control bam file> -f BAM -g hs --keep-dup all --nomodel --shift -15 --extsize 50 -B --call-summits --llocal 0 --slocal 0 --fe-cutoff 3 -q 0.05). The resulting peak files from each strand were merged.

Homer (Heinz *et al.*, 2010) was used to perform motif enrichment analysis both for de novo motifs and known motifs (findMotifsGenome.pl parameters: <peaks bed file> hg19 <output directory> -mknown<motifs file.motifs> -p 6 -size given -norevopp -len 6,7,8,9,10). De novo motif analysis identified several motifs highly similar to the published QKI motif which were highly enriched (p-value: <<1e-100) and found in a large proportion of the peaks.

TCGA splicing data analysis

Relationship between QKI expression and gene splicing in TCGA cancer samples

On 28/01/2016, the Broad GDAC Firehose website (http://gdac.broadinstitute.org/) was used to obtain publicly available (Level 3) gene expression and splice junction read count data, derived from RNA-seq (Illumina HiSeq, 'RNASeqV2' pipeline), from The Cancer Genome Atlas (TCGA), for each of seven cancers (Breast Invasive Carcinoma: BRCA; Prostate Adenocarcinoma: PRAD; Bladder Urothelial Carcinoma: BLCA, Pan-Kidney

(Kidney Chromophobe, Kidney Renal Clear Cell Carcinoma and Kidney Renal Papillary Cell Carcinoma): KIPAN, Lung Adenocarcinoma: LUAD, Lung Squamous Cell Carcinoma: LUSC, Stomach Adenocarcinoma and Esophageal Carcinoma: STES). For example, file names matching '*unc.edu.*.rsem.genes.normalized_results*' were used for gene expression and '**junction_quantification.txt*' were used for splice junction analysis. For each of these cancers, data from at least 450 samples were available.

To analyse the relationship between differential splicing and QKI expression, the reads supporting a particular splice junction were expressed as a percentage of the total number of reads for any junction using the same 5' (or 3') splice site. This is a generalised form of PSI, a metric that is commonly used for analysis of simple splicing events. Source code for this analysis is available in the repository at https://bitbucket.org/sacgf/2018_pillman_QKI_splicing_in_EMT.

The analysis was performed as follows:

1. Assign reads to genes: the number of reads supporting each junction was obtained from the resulting splice junction files and for each junction, the parent gene(s) were deduced as follows: first, if an identical junction could be identified in a UCSC transcript, the corresponding gene name was used; otherwise, the gene name(s) of overlapping transcripts on the same strand were used. The analyses were restricted to only splice junctions that were unambiguously assigned to a single gene.

2. Calculate the percentage of splicing across the junction: the total number and percentage of reads was calculated separately for 5' and 3' ends of every junction as follows: firstly, read counts were regularised by adding a pseudocount of 0.1. Then, for both 5' and 3' ends of the junction, groups of two or more junctions were identified which shared an end position. For each junction in a group, the number of reads which cross the junction was expressed as a percentage of the total number of reads across any junction in the group (e.g. if junctions A, B and C share a 5' end site and 20, 10 and 70 reads map across these junctions, respectively, then $PercentSpliced_A$ = 20%, $PercentSpliced_B$ = 10% and $PercentSpliced_C$ = 70%). This was performed separately for each sample. Loci which had a) had very low expression in all samples (<10 reads), or b) where the expression of one isoform dominated (>92% of reads in one isoform in all samples) were not included in further analysis.

3. Analyse relationship between QKI expression and alternative splicing: QKI expression was obtained from RSEM normalised gene-level expression data and samples were filtered to exclude samples without both gene expression and splicing data. To prevent spurious results from low read or sample counts, splice junctions were filtered, requiring at least moderate expression at the locus (more than 10 reads in the group) in at least 100 samples. A Welch's t-test (for independent samples of unequal variance) was used to compare the splicing percentages for the samples with highest and lowest expression of QKI, selecting for each group, either 10% of the total number of samples or 50 samples, whichever was fewer. Multiple testing correction was performed using the Benjamini/Hochberg method (Python statsmodels package, multicomp.multipletests function) with statistical significance level (family-wise error rate) of 0.05. The results were visualised by plotting QKI expression levels (x-axis) against the splicing for each splice junction and 5' or 3' end, marking the samples used for the statistical comparison.

Pan-cancer splicing analysis

To ensure only the highest-confidence splicing changes were included, genes were filtered by the statistical significance of the QKI-related splicing change (p-value from the Welch's t-test) with more stringent cutoffs being used for cancers with larger numbers of samples and/or large differences QKI expression between samples: BLCA and STES ($p<0.001$), PRAD, LUSC, LUAD ($p < 0.0001$) and BRCA and KIPAN ($p<0.00001$). Loci with changes in splicing of less than 10% were also excluded. Where multiple loci remained for a single gene, the locus with the largest change in PSI was used as a representative. After collating this data for all cancers, further filtering was performed to require that genes had mean differences in splicing of at least 35% in at least one cancer and were constitutively expressed at a moderate level in all cancers. This was defined as a median gene expression level across samples >500 in TCGA gene expression data (Level 3 RSEM normalised gene-level data from the RNAseqv2 pipeline on Illumina Hiseq data). The heatmap was produced using the clustermap function from the Python seaborn package, using the 'cityblock' (Manhattan) method to calculate distance and 'weighted' linkage method for hierarchical clustering.

Kaplan-Meier survival analysis

For meta-analysis on the combined cohort from KM plotter (Gyorffy *et al.*, 2010), the mean of three probes (212262_at , 212263_at and 212265_at) corresponding to the QKI-5 specific 3'UTR were used to assess distant metastasis-free survival on the combined unsegregated patient cohort. After calculation of all percentages between the upper and lower quartiles, the best performing threshold was utilised. For the Hatzis et al dataset (GSE25066) (Hatzis *et al.*, 2011) all samples were included and distant metastasis-free survival on a mean split of QKI expression (214543_x_at probe) was calculated. Statistical significance and hazard ratios were calculated using GraphPad Prism software.

qPCR and change in percentage spliced in (PSI) analysis

qPCR for splice events was performed in triplicate with primers sets that specifically amplify the exon included and excluded product using the QuantiTect SYBR green PCR kit (Qiagen) on a Rotorgene 6000 series PCR machine (Qiagen). Primer sequences are shown in Table EV8. Analysis was performed using comparative quantitation feature of the Rotorgene software with data normalised to GAPDH expression. Change in PSI was calculated by substracting the cycle threshold of excluded product from included product, converting this to fold-difference (FD), and using the formula 1/(1-FD). Data shown are normalised to d0 (HMLE + TGFβ timecourse) or mCherry (HMLE-iQKI-5 doxycycline timecourse) at a starting point of zero.

References for Appendix Supplementary Methods

Anders S, Reyes A, Huber W (2012) Detecting differential usage of exons from RNA-seq data. *Genome Res* 22: 2008-17

Gyorffy B, Lanczky A, Eklund AC, Denkert C, Budczies J, Li Q, Szallasi Z (2010) An online survival analysis tool to rapidly assess the effect of 22,277 genes on breast cancer prognosis using microarray data of 1,809 patients. *Breast cancer research and treatment* 123: 725-31

Hatzis C, Pusztai L, Valero V, Booser DJ, Esserman L, Lluch A, Vidaurre T, Holmes F, Souchon E, Wang H, Martin M, Cotrina J, Gomez H, Hubbard R, Chacon JI, Ferrer-Lozano J, Dyer R, Buxton M, Gong Y, Wu Y et al. (2011) A genomic predictor of response and survival following taxane-anthracycline chemotherapy for invasive breast cancer. *JAMA* 305: 1873-81

Heinz S, Benner C, Spann N, Bertolino E, Lin YC, Laslo P, Cheng JX, Murre C, Singh H, Glass CK (2010) Simple combinations of lineage-determining transcription factors prime cis-regulatory elements required for macrophage and B cell identities. *Mol Cell* 38: 576-89

Jensen KB, Darnell RB (2008) CLIP: crosslinking and immunoprecipitation of in vivo RNA targets of RNA-binding proteins. *Methods Mol Biol* 488: 85-98

Kim D, Pertea G, Trapnell C, Pimentel H, Kelley R, Salzberg SL (2013) TopHat2: accurate alignment of transcriptomes in the presence of insertions, deletions and gene fusions. *Genome Biol* 14: R36

Li H, Handsaker B, Wysoker A, Fennell T, Ruan J, Homer N, Marth G, Abecasis G, Durbin R, Genome Project Data Processing S (2009) The Sequence Alignment/Map format and SAMtools. *Bioinformatics* 25: 2078-9

Liu R, Loraine AE, Dickerson JA (2014) Comparisons of computational methods for differential alternative splicing detection using RNA-seq in plant systems. *BMC Bioinformatics* 15: 364

Martin M (2011) Cutadapt Removes Adapter Sequences From High-Throughput Sequencing Reads. *EMBnetjournal* 17: 10-12

Shen S, Park JW, Lu ZX, Lin L, Henry MD, Wu YN, Zhou Q, Xing Y (2014) rMATS: robust and flexible detection of differential alternative splicing from replicate RNA-Seq data. *Proc Natl Acad Sci U S A* 111: E5593-601

Sutandy FX, Hildebrandt A, Konig J (2016) Profiling the Binding Sites of RNA-Binding Proteins with Nucleotide Resolution Using iCLIP. *Methods Mol Biol* 1358: 175-95

Trapnell C, Hendrickson DG, Sauvageau M, Goff L, Rinn JL, Pachter L (2013) Differential analysis of gene regulation at transcript resolution with RNA-seq. *Nat Biotechnol* 31: 46-53

Van Nostrand EL, Pratt GA, Shishkin AA, Gelboin-Burkhart C, Fang MY, Sundararaman B, Blue SM, Nguyen TB, Surka C, Elkins K, Stanton R, Rigo F, Guttman M, Yeo GW (2016) Robust transcriptome-wide discovery of RNA-binding protein binding sites with enhanced CLIP (eCLIP). *Nat Methods* 13: 508-14

Zhang Y, Liu T, Meyer CA, Eeckhoute J, Johnson DS, Bernstein BE, Nusbaum C, Myers RM, Brown M, Li W, Liu XS (2008) Model-based analysis of ChIP-Seq (MACS). *Genome Biol* 9: R137

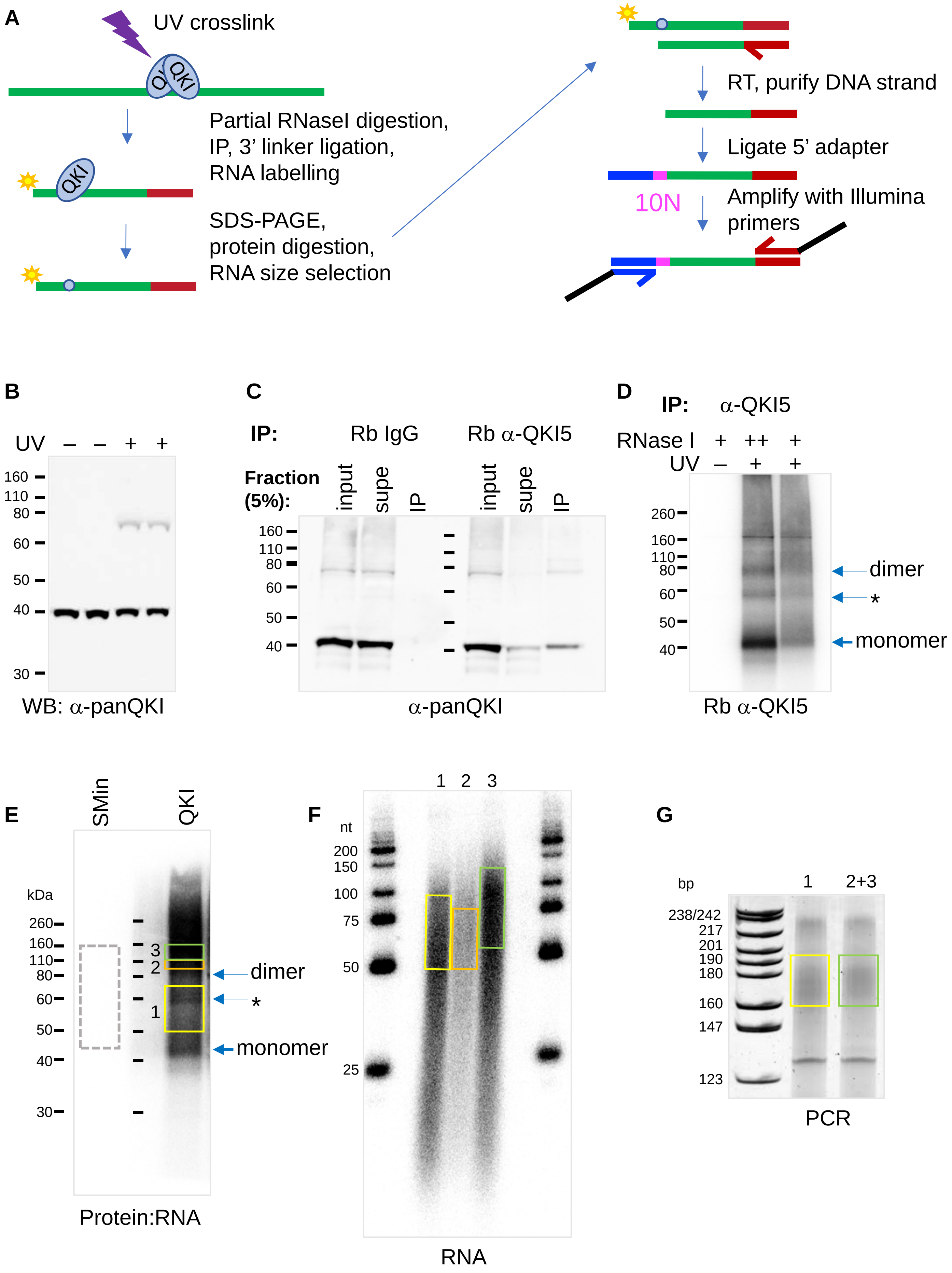
A
UV crosslink
QKI
Partial RNaseI digestion,
IP, 3' linker ligation,
RNA labelling
SDS-PAGE,
protein digestion,
RNA size selection
RT, purify DNA strand
Ligate 5' adapter
10N
Amplify with Illumina
primers
B
UV – – + +
160
110
80
60
50
40
30
WB: α-panQKI
C
IP: Rb IgG Rb α-QKI5
Fraction (5%):
input
supe
IP
160
110
80
60
50
40
α-panQKI
D
IP: α-QKI5
RNase I + ++ +
UV – + +
260
160
110
80
60
50
40
dimer
*
monomer
Rb α-QKI5
E
SMin
QKI
kDa
260
160
110
80
60
50
40
30
3
2
1
dimer
*
monomer
Protein:RNA
F
1 2 3
nt
200
150
100
75
50
25
RNA
G
bp
1 2+3
238/242
217
201
190
180
160
147
123
PCR

Appendix Supplementary Methods Figure S1 – QKI-5 HITS-CLIP. (A) Schematic representation of HITS-CLIP procedure. **(B)** QKI Western blot indicating the migration of QKI monomer and UV-crosslink dependent presumptive QKI dimer. **(C)** Western blot confirming specificity of QKI IP compared to IgG control. **(D)** Radiolabelled RNA:protein complexes following IP, SDS-PAGE and transfer to nitrocellulose. No signal is seen in the absence of UV crosslinking; high RNAse treatment indicates the position of QKI monomer, dimer and co-purifying unknown species (*); low RNase concentration elicits a smear up the gel of QKI plus crosslinked RNA fragments. **(E)** Preparative SDS-PAGE showing excision of QKI-RNA complexes. Regions were selected to capture 3'-linker-ligated, QKI-associated radiolabelled RNAs for both monomeric and dimeric quaking, whilst avoiding RNA associated with the unknown co-purifying protein. The corresponding region excised from unlabelled SMin (minus 3' adapter) is also shown. **(F)** Size selection of QKI-associated radiolabelled RNA fragments of the correct predicted size by denaturing polyacrylamide gel. Numbers correspond to protein:RNA complexes excised in (E). **(G)** Gel purification of QKI HITS-CLIP PCR-amplified libraries.

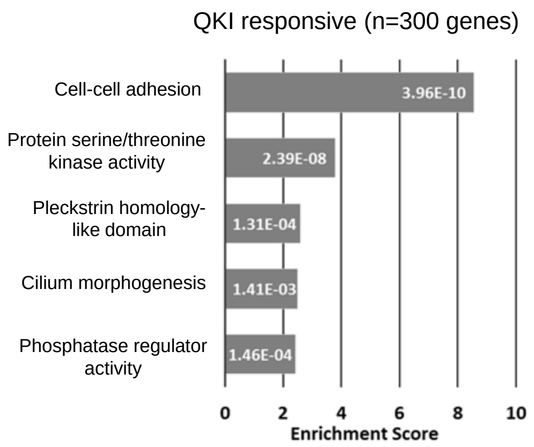
QKI responsive (n=300 genes)
Cell-cell adhesion
Protein serine/threonine kinase activity
Pleckstrin homology-like domain
Cilium morphogenesis
Phosphatase regulator activity
3.96E-10
2.39E-08
1.31E-04
1.41E-03
1.46E-04
0
2
4
6
8
10
Enrichment Score

Appendix Figure S1: Gene ontology analysis of QKI-5 regulated spliced genes during EMT The top five functional annotations derived from GO analysis of the 300 most significant genes whose change in splicing during EMT was reversed by knockdown of QKI-5 are ranked by enrichment score with lowest p-values are indicated.

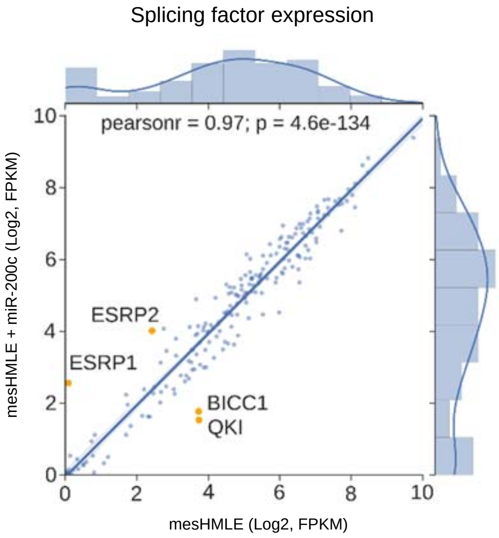
Splicing factor expression
pearsonr = 0.97; p = 4.6e-134
ESRP2
ESRP1
BICC1
QKI
mesHMLE + miR-200c (Log2, FPKM)
mesHMLE (Log2, FPKM)
0
2
4
6
8
10

Appendix Figure S2: Expression of splicing factors in response to miR-200c transfection

Differential expression of 224 known or putative splicing factors (Han *et al.*, 2013) in mesHMLE cells transfected with miR-200c compared with control. Highly up- and down-regulated genes are highlighted.

References for Appendix Figure S2

Han H, Irimia M, Ross PJ, Sung HK, Alipanahi B, David L, Golipour A, Gabut M, Michael IP, Nachman EN, Wang E, Trcka D, Thompson T, O'Hanlon D, Slobodeniuc V, Barbosa-Morais NL, Burge CB, Moffat J, Frey BJ, Nagy A et al. (2013) MBNL proteins repress ES-cell-specific alternative splicing and reprogramming. *Nature* 498: 241-5
