# Supplementary material for: miR‐200/375 control epithelial plasticity‐associated alternative splicing by repressing the RNA‐binding protein Quaking
Source: EMBO J. 2018 Jun 6;37(13):e99016. doi: 10.15252/embj.201899016 (PMC6028027; doi:10.15252/embj.201899016)
Supplement: Supplementary file 2 — Expanded View Figures PDF [file EMBJ-37-e99016-s002.pdf]

Expanded View Figures

Figure EV1. QKI expression in clinical data sets.

A Scatterplot of QKI versus miR-200c expression in cancer data sets with Pearson correlation coefficients and associated *P*-values indicated.
B Kaplan–Meier survival analysis showing distant metastasis-free survival (DMFS) from breast cancer in unsegregated combined cohort data from KM plotter (Gyorffy *et al*, 2010) and the Hatzis *et al* (GSE25066) (Hatzis *et al*, 2011) data sets. Hazard ratios (HR) and *P*-values are indicated.
C Relative expression of QKI in tumour subtypes of the TCGA breast cancer data set displayed as a minimum-to-maximum box-and-whisker plot. Box limits represent the 25th–75th percentiles with a median central line. Whiskers extend to the minimum and maximum values with all data points shown. Significance between each subtype was calculated by two-tailed unpaired *t*-tests.
D Relative expression of QKI in prostate cancers exhibiting biochemical recurrence compared with non-recurrence in the TCGA prostate cancer data set displayed as a minimum-to-maximum box-and-whisker plot. Significance was calculated by a two-tailed unpaired *t*-test.
E Scatterplot of QKI versus miR-375 expression in cancer data sets with Pearson correlation coefficients and associated *P*-values indicated.

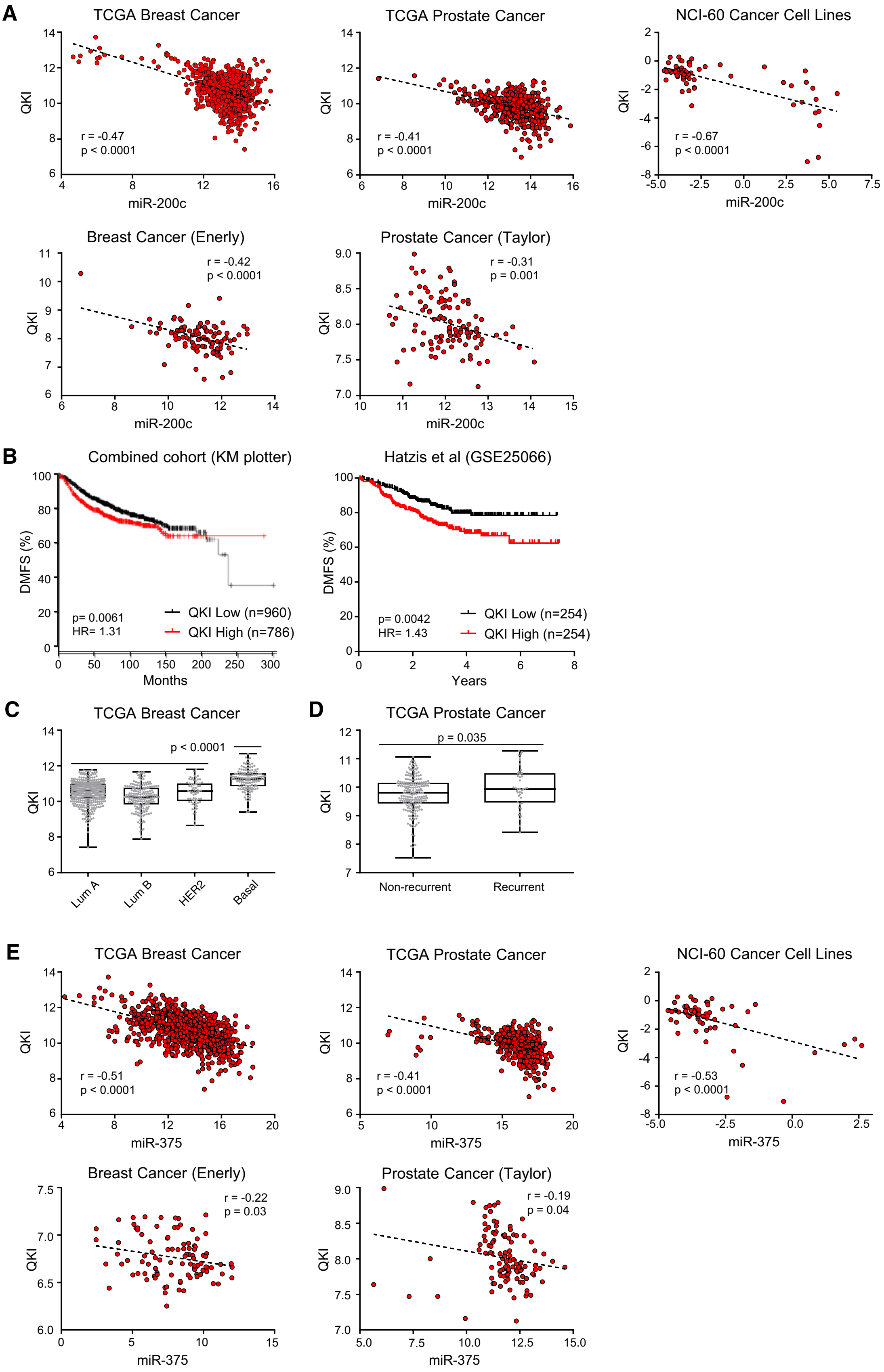

Figure EV1.

© 2018 The Authors

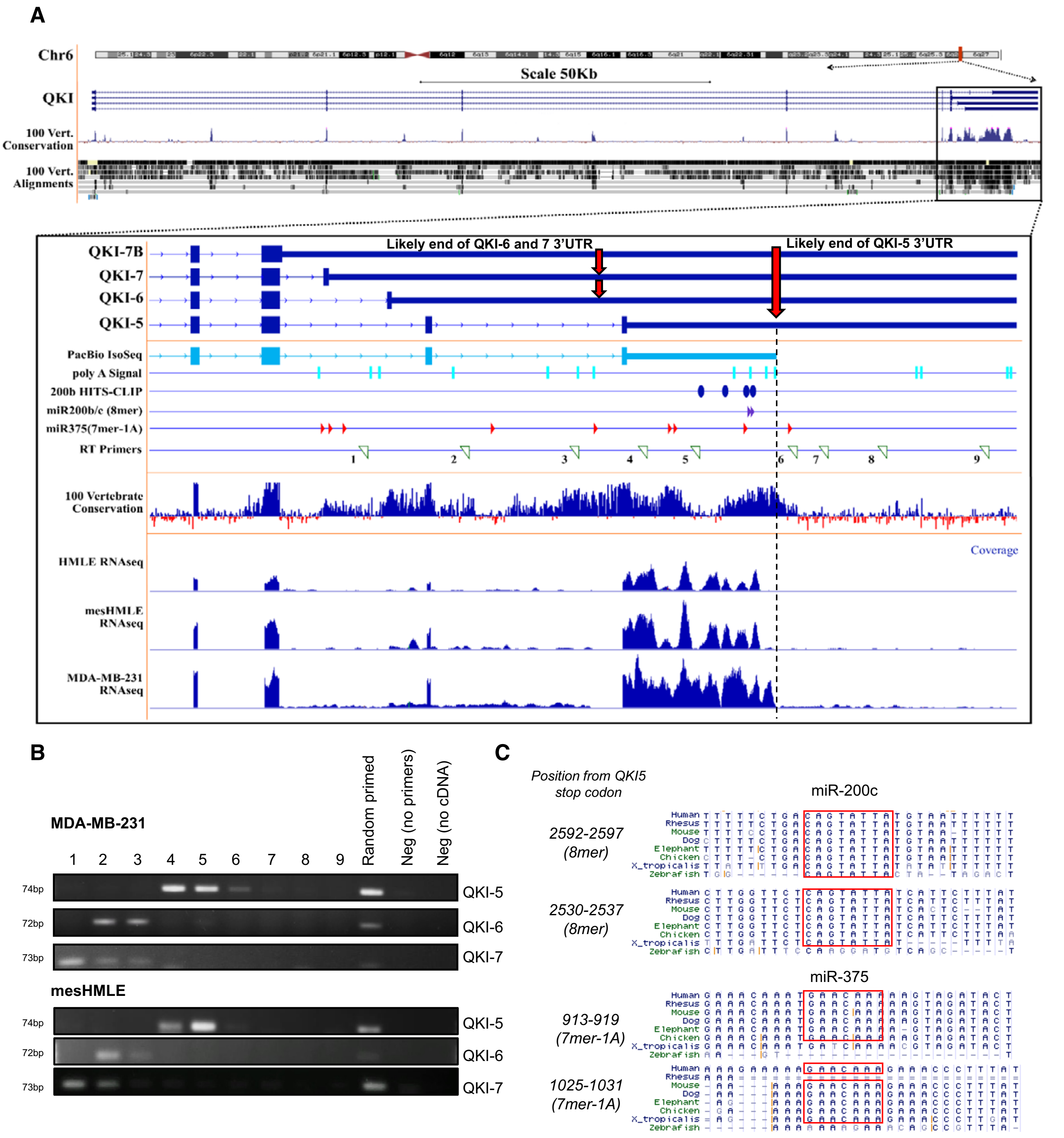

Figure EV2. Mapping of the QKI locus 3′UTRs.

A The full QKI gene locus is shown in the upper panel, with the divergent C-terminal end and 3′UTRs for each isoform magnified below. Track names for each major isoform are indicated. Annotation of a full-length read from a PacBio IsoSeq experiment (Pacific Biosciences), and sequencing coverage reads from in-house HMLE, mesHMLE and MDA-MB-231 cells are shown, which terminate in close proximity to the decline of high sequence conservation. Putative locations of polyA signal sequences, miR-200b/c (8mer) and miR-375 (7mer-1A) sites are shown. The locations of miR-200b HITS-CLIP peaks (Bracken *et al*, 2014) and RT primers used for priming cDNA synthesis are indicated.

B QKI-5, QKI-6 or QKI-7 isoform PCR on cDNA synthesised using the specific RT primers numbered in (A) using MDA-MB-231 and mesHMLE RNA.

C Location of functional miR-200c and miR-375 binding sites showing their cross-species conservation. The red box indicates the miRNA binding site complementary to the seed sequence.

Source data are available online for this figure.

© 2018 The Authors

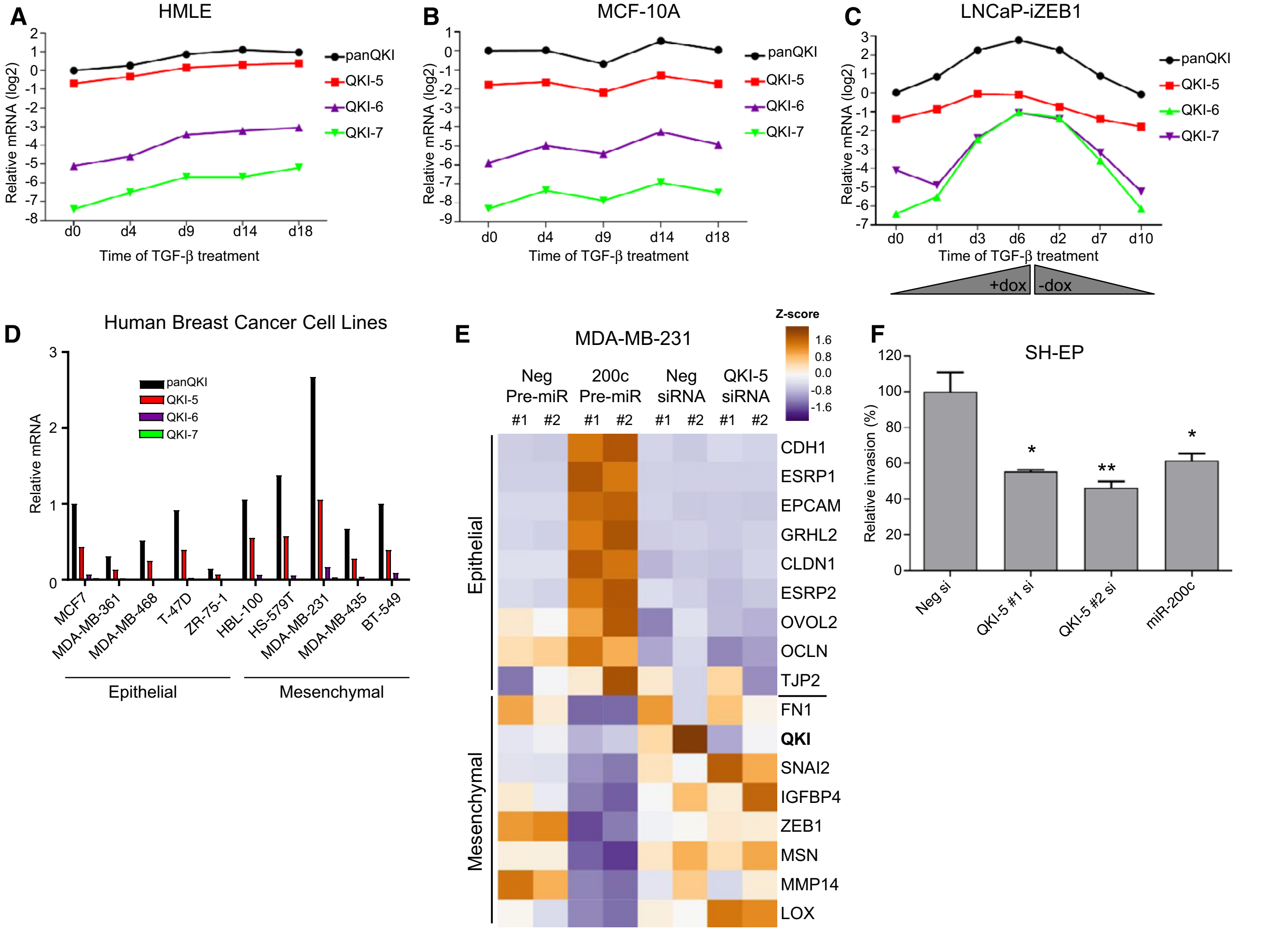

Figure EV3. Expression and function of QKI in cancer cell lines.

A–D Quantitative PCR showing QKI-5, QKI-6 and QKI-7 or total QKI levels (panQKI) across the EMT timecourses and breast cancer cell lines described in Fig 2.

E Heat map of microarray data showing a subset of EMT genes following transfection of MDA-MB-231 cells with miR-200c, QKI-5 siRNA or negative controls.

F Invasion assay following QKI-5-specific siRNA knockdown or miR-200c transfection in SH-EP cells. Experiments were performed with three biological replicates and are shown as mean ± SEM. Significance was measured by two-tailed unpaired *t*-tests. $*P < 0.05$ and $**P < 0.01$.

Figure EV4. Transcriptome changes mediated by QKI-5 during EMT.

A Volcano plots showing differential expression during EMT (HMLE versus mesHMLE), in mesHMLE following QKI-5 knockdown, or mesHMLE following introduction of miR-200c. Numbers of significant genes (above dotted line with q-value < 0.05) are shown, with genes having > 2-fold differential expression highlighted in blue (downregulated) or red (upregulated).

B Venn diagram showing the overlap of genes in mesHMLE and MDA-MB-231 cells that are regulated during EMT and by knockdown of QKI-5 (fold change > 2, $P < 0.05$).

C Heat map of genes that significantly change in MDA-MB-231 cells in response to miR-200b (microarray data) with corresponding changes in the same genes in mesHMLE cells in response to miR-200c (RNA-seq) shown ($n = 679$).

D Venn diagram showing the overlap of skipped or included exon events during EMT or in response to knockdown of QKI-5 in mesHMLE cells ($\Delta PSI > 5\%$, FDR < 0.05).

E Pie chart of the relative proportions of QKI-5 HITS-CLIP peaks in genomic regions.

F De novo motif enrichment in QKI-5 HITS-CLIP peaks, as reported by findMotifsGenome.pl from the Homer package (Heinz *et al*, 2010), relative to a background of introns surrounding constitutively expressed exons during EMT.

G Relative position of QKI motifs in QKI-5 HITS-CLIP peaks on the sense strand compared to anti-sense strand (background).

 © 2018 The Authors

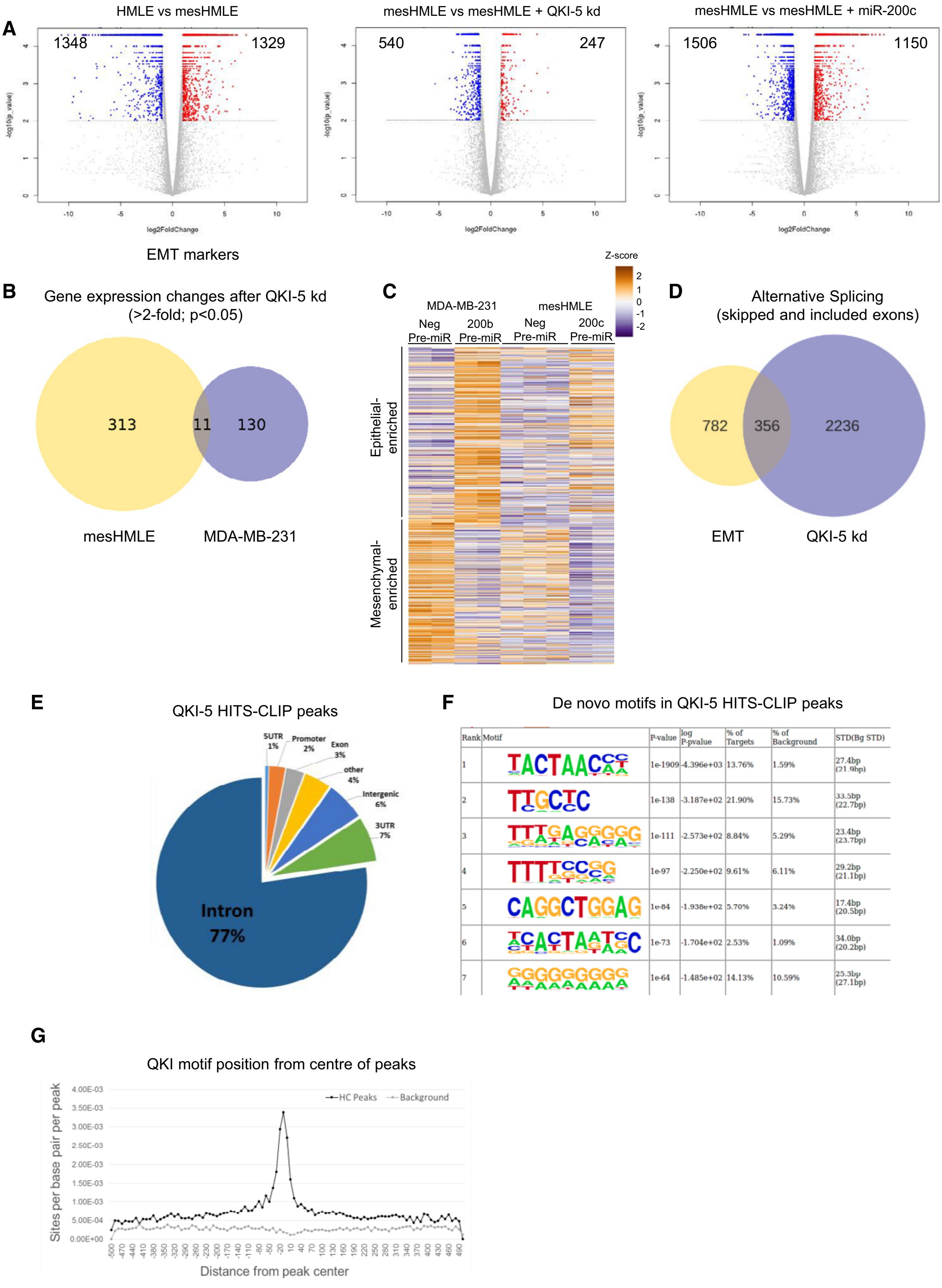
A
HMLE vs mesHMLE
1348
1329
mesHMLE vs mesHMLE + QKI-5 kd
540
247
mesHMLE vs mesHMLE + miR-200c
1506
1150
-log10(p_value)
log2FoldChange
EMT markers
B
Gene expression changes after QKI-5 kd
(>2-fold; p<0.05)
313
11
130
mesHMLE
MDA-MB-231
C
Z-score
MDA-MB-231
mesHMLE
Neg Pre-miR
200b Pre-miR
Neg Pre-miR
200c Pre-miR
Epithelial-enriched
Mesenchymal-enriched
D
Alternative Splicing
(skipped and included exons)
782
356
2236
EMT
QKI-5 kd
E
QKI-5 HITS-CLIP peaks
5UTR 1%
Promoter 2%
Exon 3%
other 4%
Intergenic 6%
3UTR 7%
Intron 77%
F
De novo motifs in QKI-5 HITS-CLIP peaks
Rank Motif P-value log P-pvalue % of Targets % of Background STD(Bg STD)
1 1e-1909 -4.396e+03 13.76% 1.59% 27.4bp (21.9bp)
2 1e-138 -3.187e+02 21.90% 15.73% 33.5bp (22.7bp)
3 1e-111 -2.573e+02 8.84% 5.29% 23.4bp (23.7bp)
4 1e-97 -2.250e+02 9.61% 6.11% 29.2bp (21.1bp)
5 1e-84 -1.938e+02 5.70% 3.24% 17.4bp (20.5bp)
6 1e-73 -1.704e+02 2.53% 1.09% 34.0bp (20.2bp)
7 1e-64 -1.485e+02 14.13% 10.59% 25.3bp (27.1bp)
G
QKI motif position from centre of peaks
HC Peaks
Background
Sites per base pair per peak
Distance from peak center

Figure EV4.

Figure EV5. Major EMT alternative splicing events regulated by the miR-200/375–QKI-5 axis.

A Sashimi plots showing percentage spliced in (PSI) of alternatively spliced events regulated by QKI-5 in HMLE cells (purple), mesHMLE cells (green) and mesHMLE cells with QKI-5 knocked down (red). Below in black are the coverage tracks of QKI-5 binding from the mesHMLE QKI-5 HITS-CLIP analysis, with the alternative exon shown in red.

B PCR of skipped exon events from (A) following transfection of MDA-MB-231 cells with miRNAs or QKI-5 siRNAs.

C Change in PSI as measured by qPCR is shown for representative splice events following TGF-β induction of EMT in HMLE cells transfected with control or QKI-5 siRNAs.

D Western blot of EMT markers and PCR of QKI-regulated alternative splicing events are shown following doxycycline induction of QKI-5 and subsequent withdrawal in HMLE-iQKI-5 cells. mCherry-expressing cell lines shown as an additional control. In the middle panel, quantitation of mRNAs by qPCR in this timecourse is shown. In the lower panel, change in PSI as measured by qPCR is shown for representative splice events in this timecourse.

Source data are available online for this figure.

 © 2018 The Authors

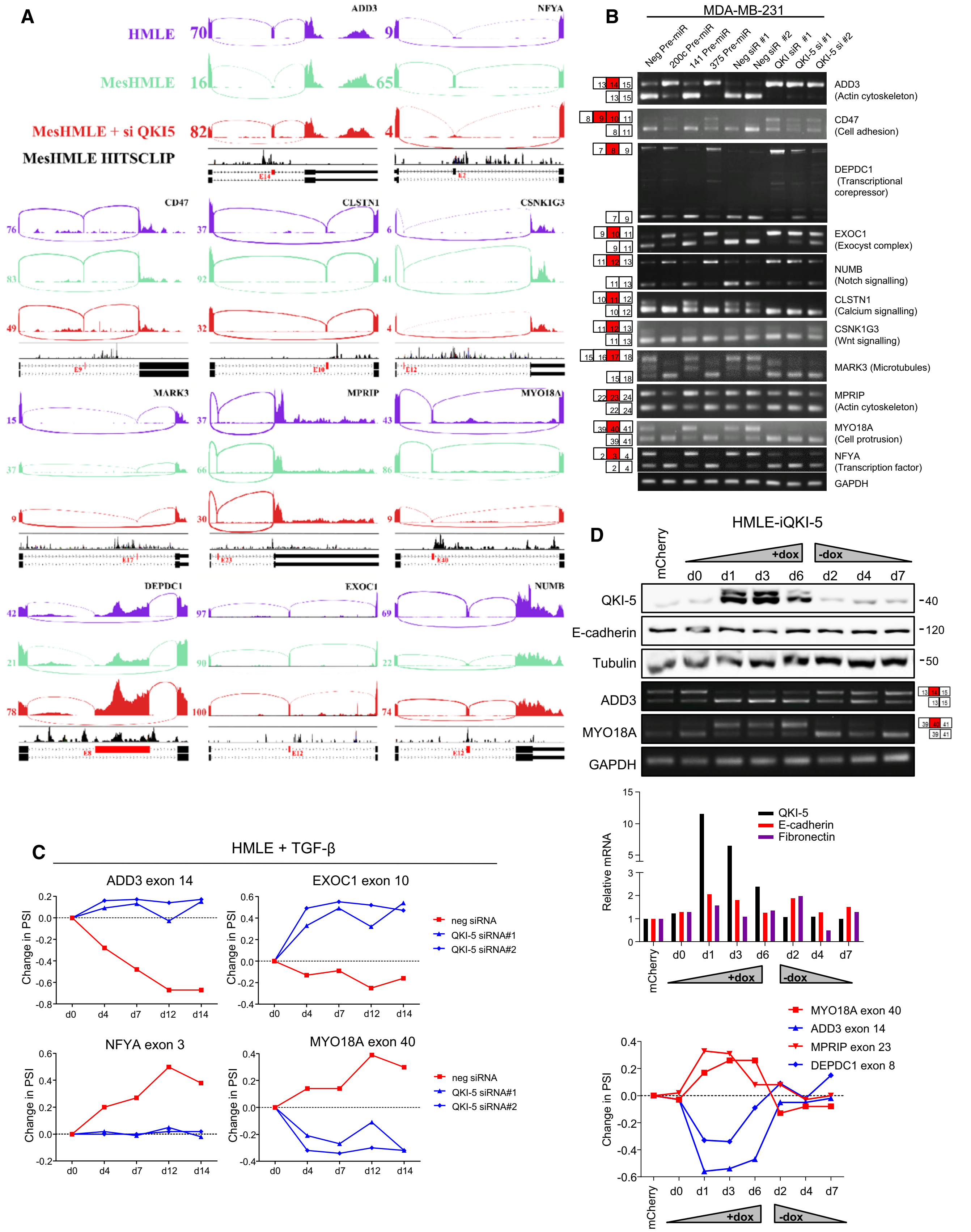

Figure EV5.

© 2018 The Authors
