# Supplementary material for: miR‐200/375 control epithelial plasticity‐associated alternative splicing by repressing the RNA‐binding protein Quaking
Source: EMBO J. 2018 Jun 6;37(13):e99016. doi: 10.15252/embj.201899016 (PMC6028027; doi:10.15252/embj.201899016)

Figure EV5 B – MDA-MB-231

Splicing PCR – Events for which QKI causes exclusion

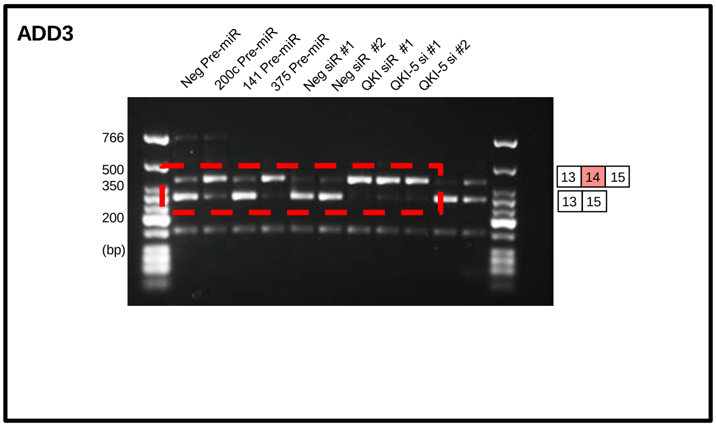

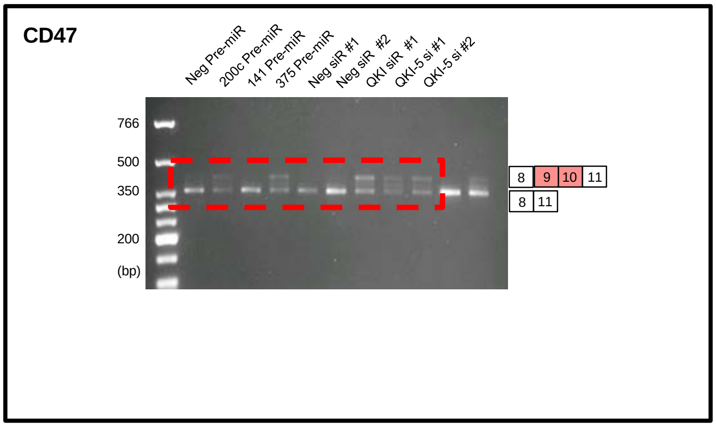

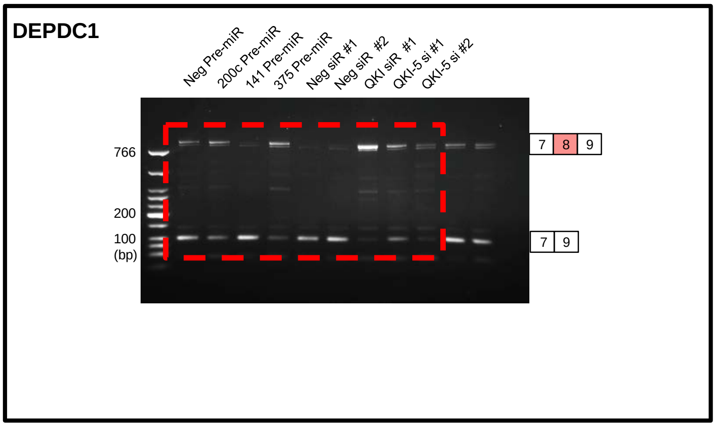

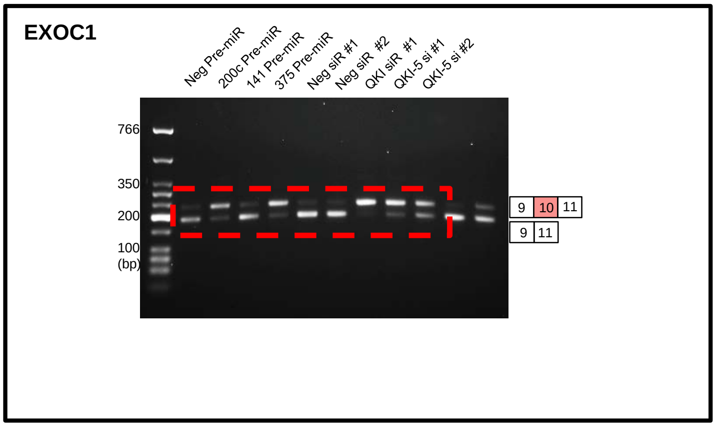

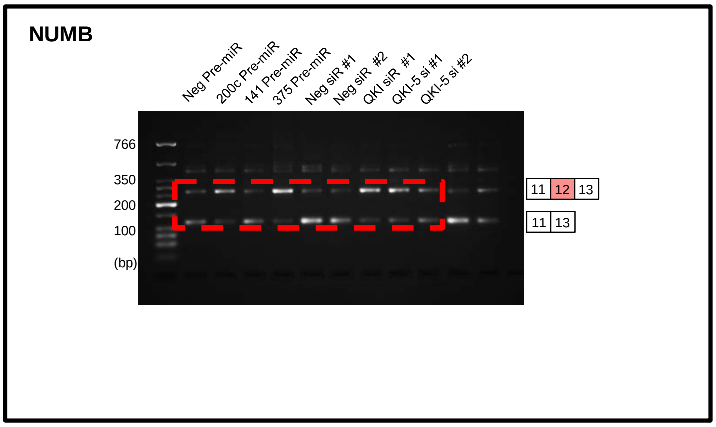

Figure EV5 B – MDA-MB-231

Splicing PCR – Events for which QKI causes inclusion

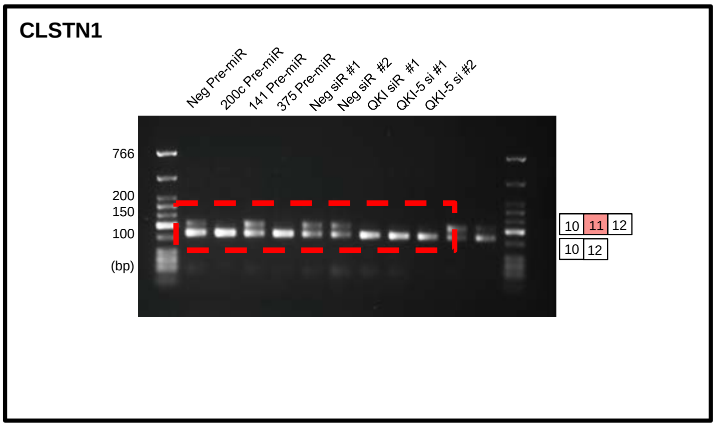

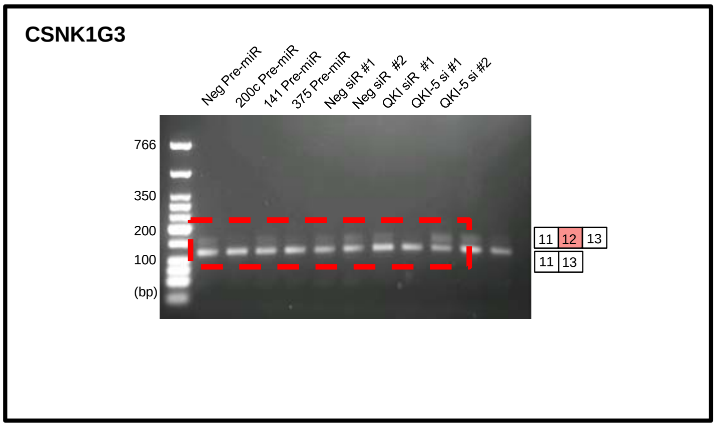

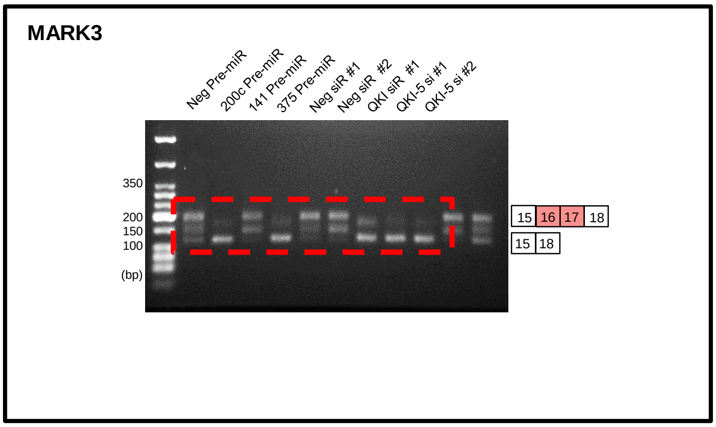

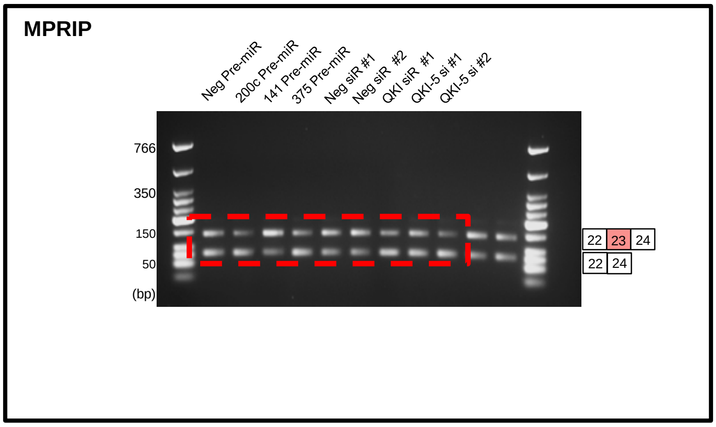

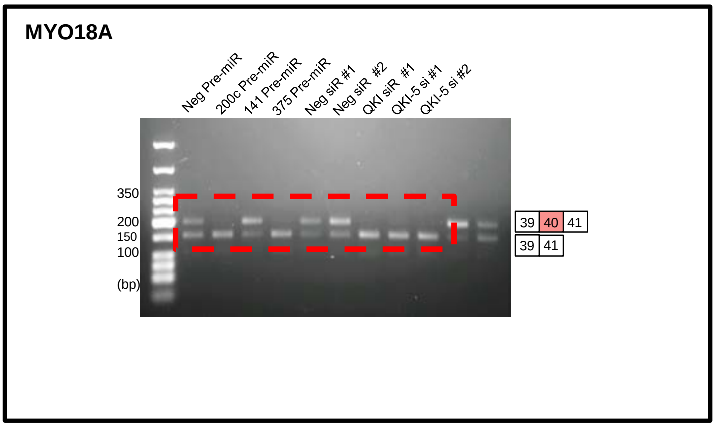

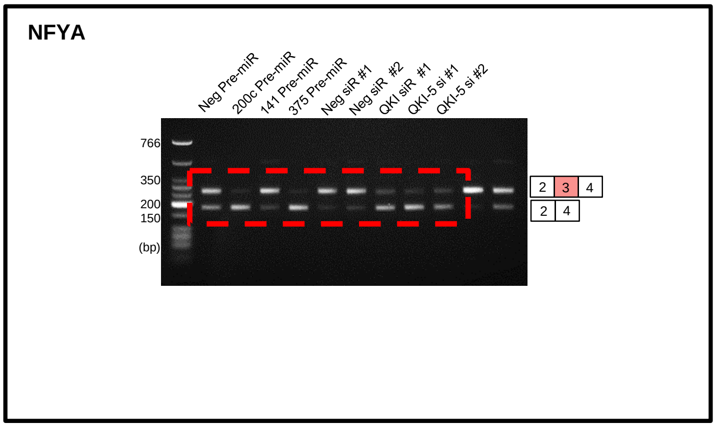

Figure EV5 B – MDA-MB-231

PCR – Normaliser

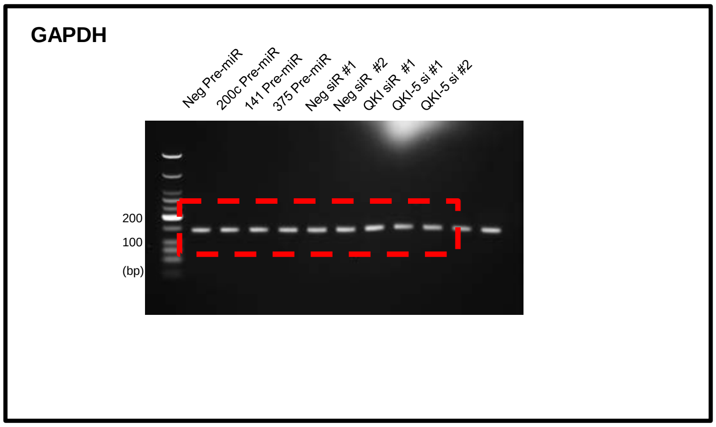

Figure EV5 D – HMLE-iQKI5

Western Blot

Splicing PCR

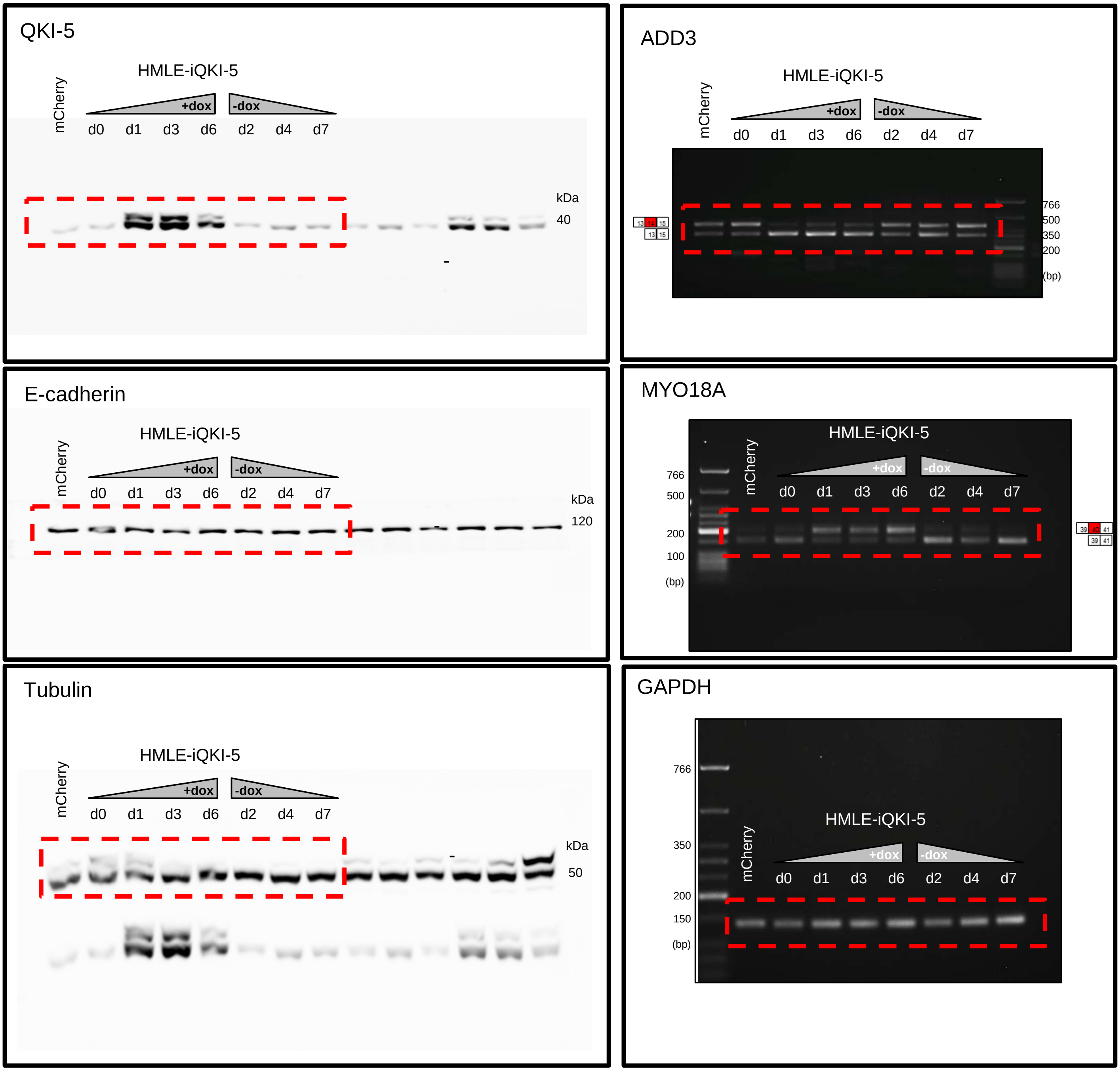

Supplement: Supplementary file 12 — Source Data for Expanded View [file EMBJ-37-e99016-s019.zip › FigEV5SD.pdf]
