# Supplementary figures and images for: miR‐200/375 control epithelial plasticity‐associated alternative splicing by repressing the RNA‐binding protein Quaking
Source: EMBO J. 2018 Jun 6;37(13):e99016. doi: 10.15252/embj.201899016 (PMC6028027; doi:10.15252/embj.201899016)

Figure EV2 B – MDA-MB-231

3'UTR Mapping PCR

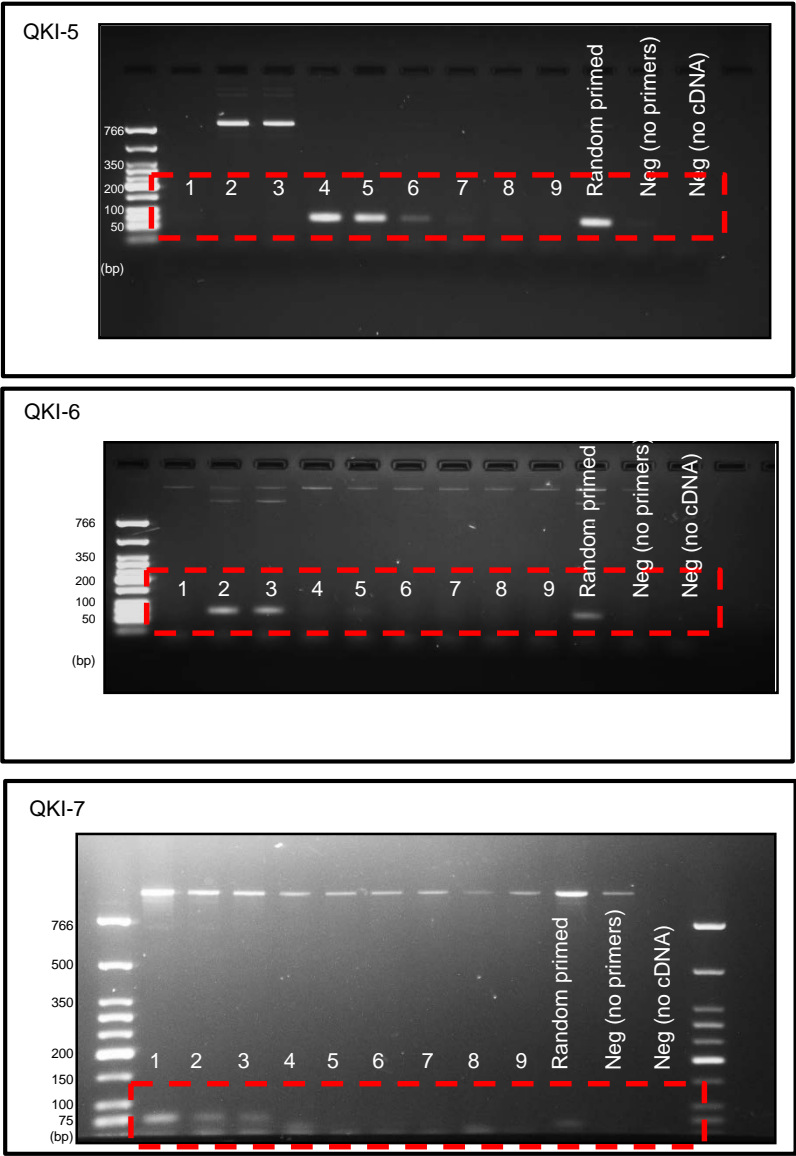

Figure EV2 B – mesHMLE

3'UTR Mapping PCR

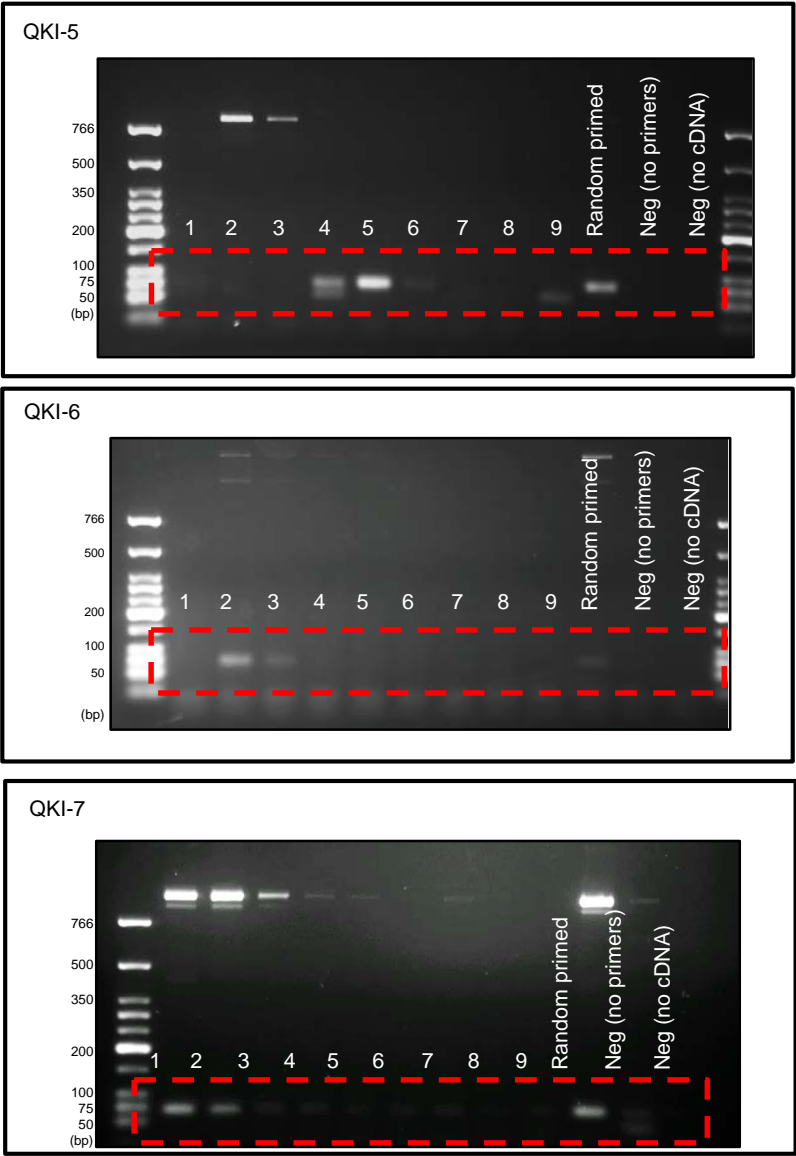

Supplement: Supplementary file 12 — Source Data for Expanded View [file EMBJ-37-e99016-s019.zip › FIgEV2SD.pdf]

Figure 1 C – Breast Cancer Panel  
Western Blots

QKI-5

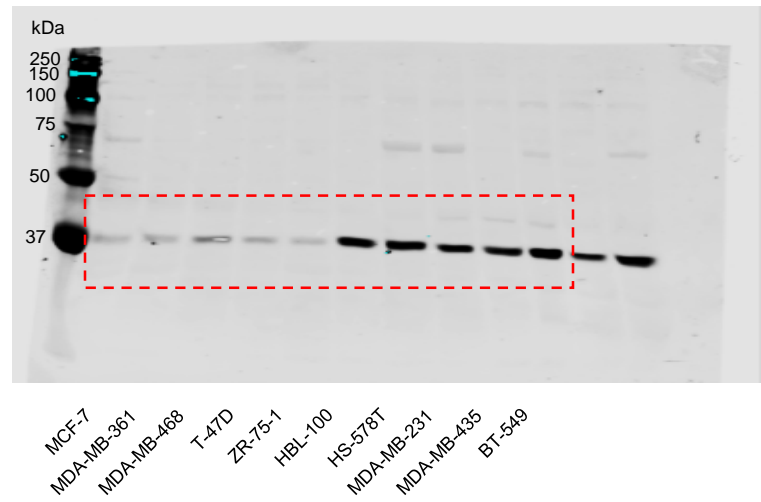

Pan-QKI

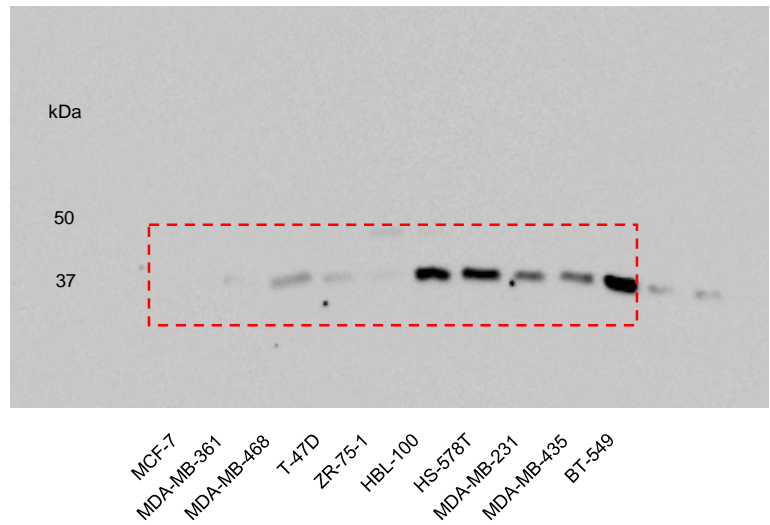

E-cadherin/Tubulin

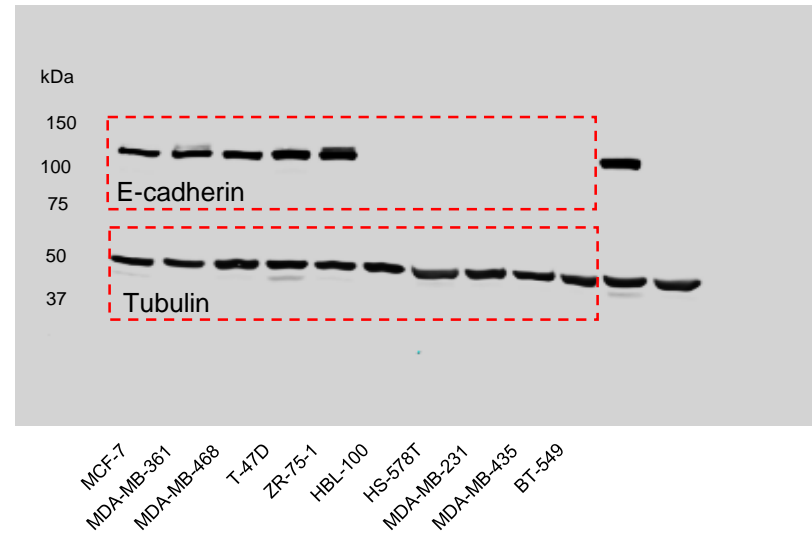

Supplement: Supplementary file 14 — Source Data for Figure 1 [file EMBJ-37-e99016-s012.pdf]

Figure 2 A - MDA-MB-231

Western Blot

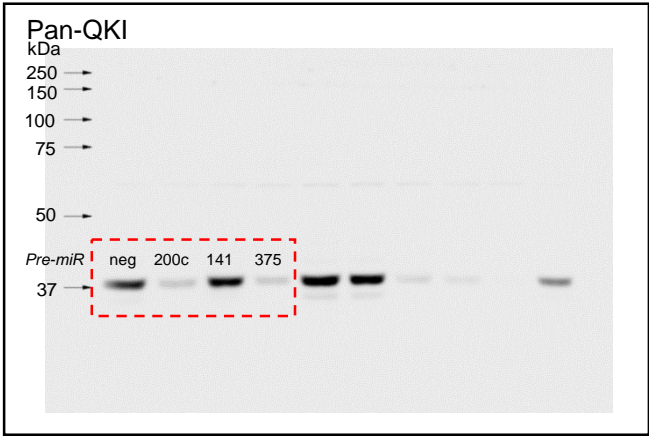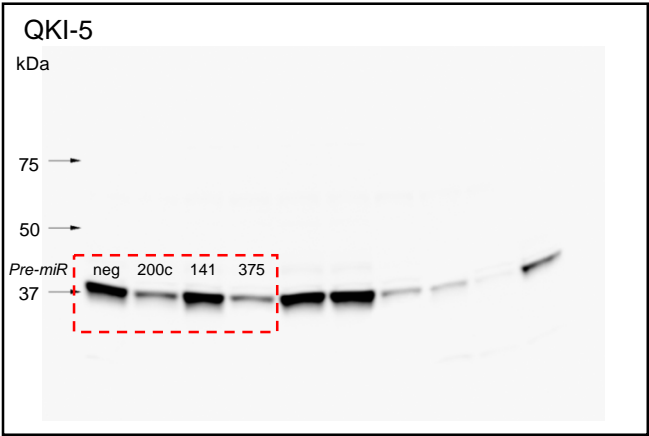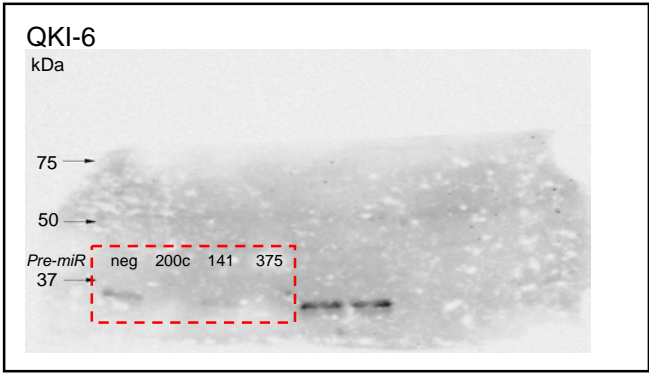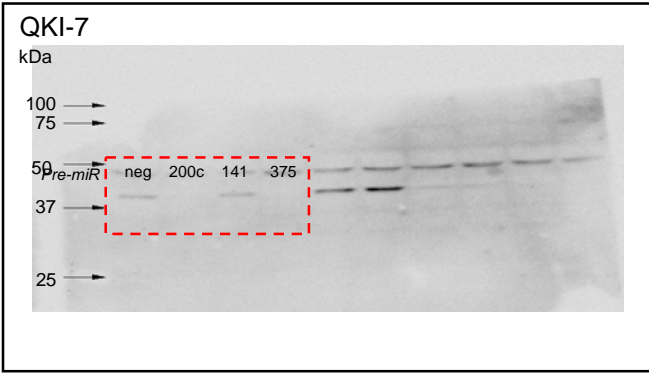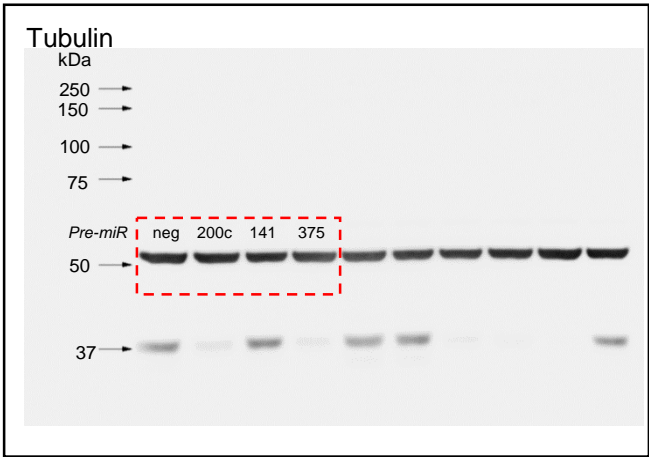

Figure 2 A - mesHMLE

Western Blot

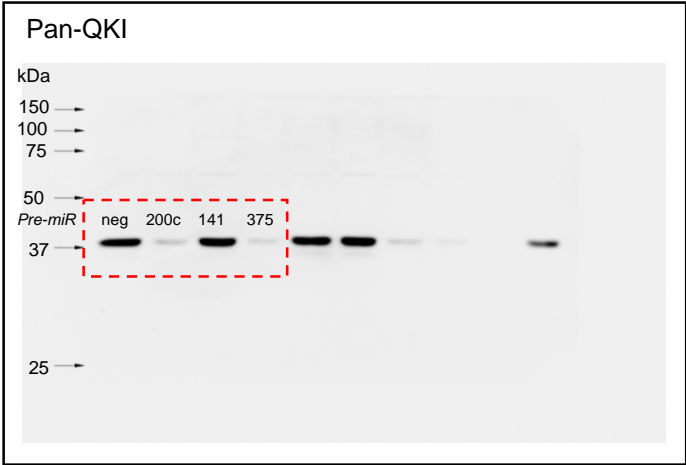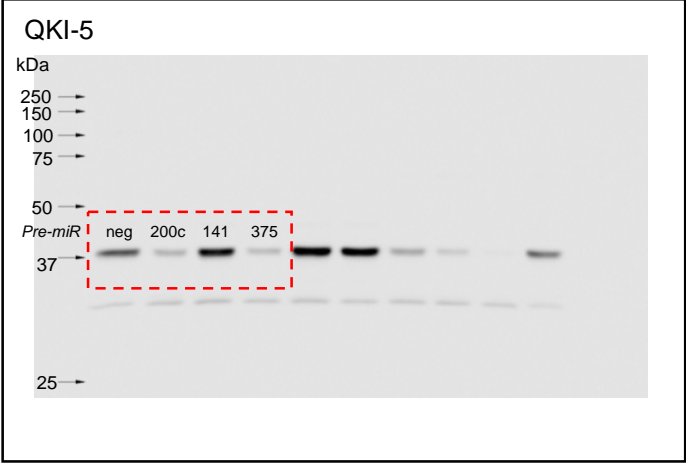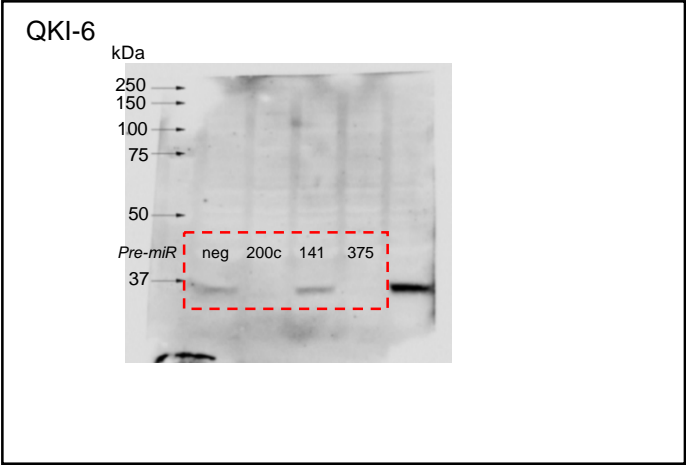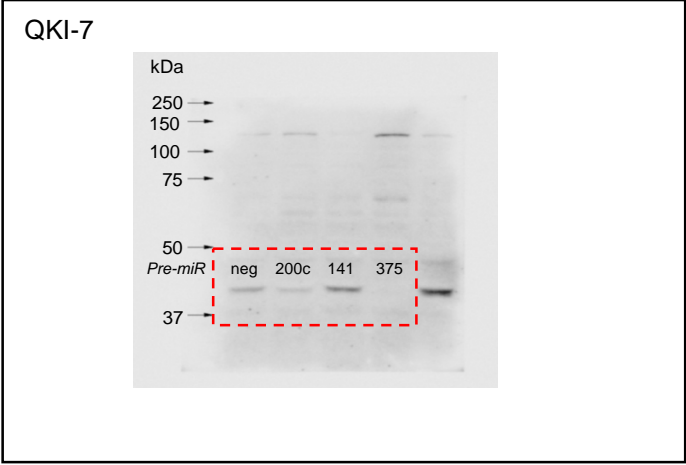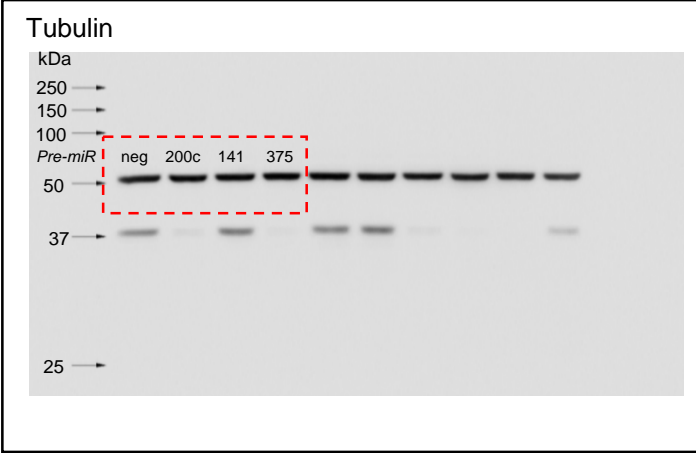

Supplement: Supplementary file 15 — Source Data for Figure 2 [file EMBJ-37-e99016-s013.pdf]

Figure 7 G

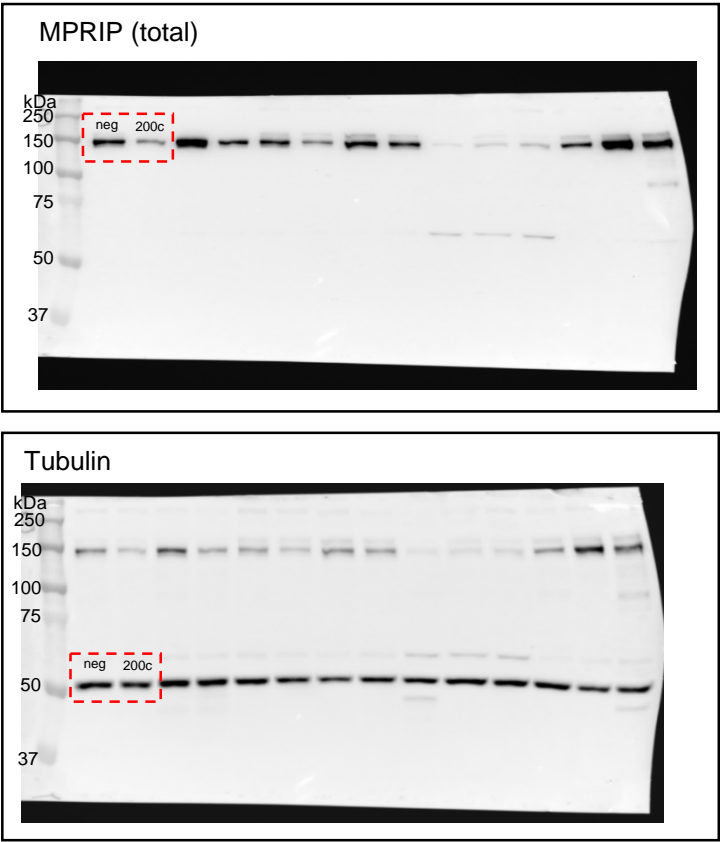

Supplement: Supplementary file 20 — Source Data for Figure 7 [file EMBJ-37-e99016-s018.pdf]
