# Supplementary material for: miR‐200/375 control epithelial plasticity‐associated alternative splicing by repressing the RNA‐binding protein Quaking
Source: EMBO J. 2018 Jun 6;37(13):e99016. doi: 10.15252/embj.201899016 (PMC6028027; doi:10.15252/embj.201899016)

# Figure 3 A - MDCK

Western Blot

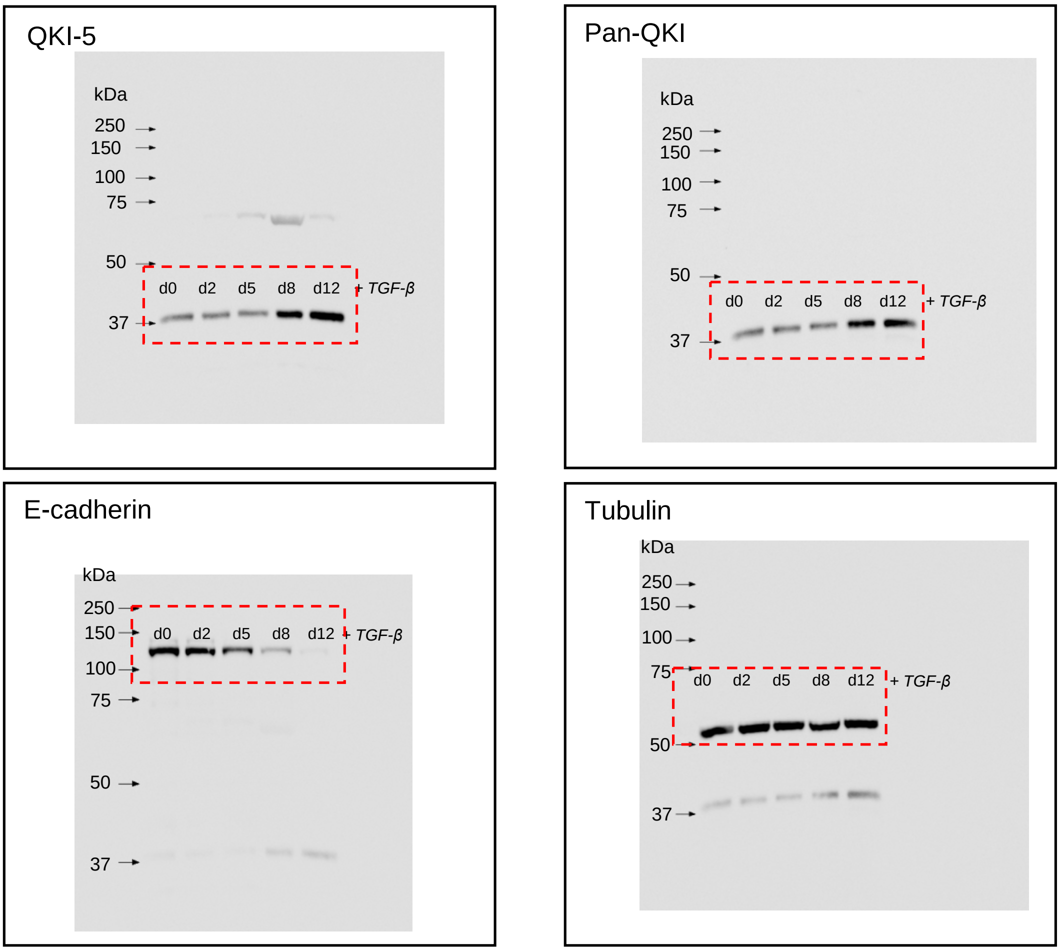

# Figure 3 A - HMLE

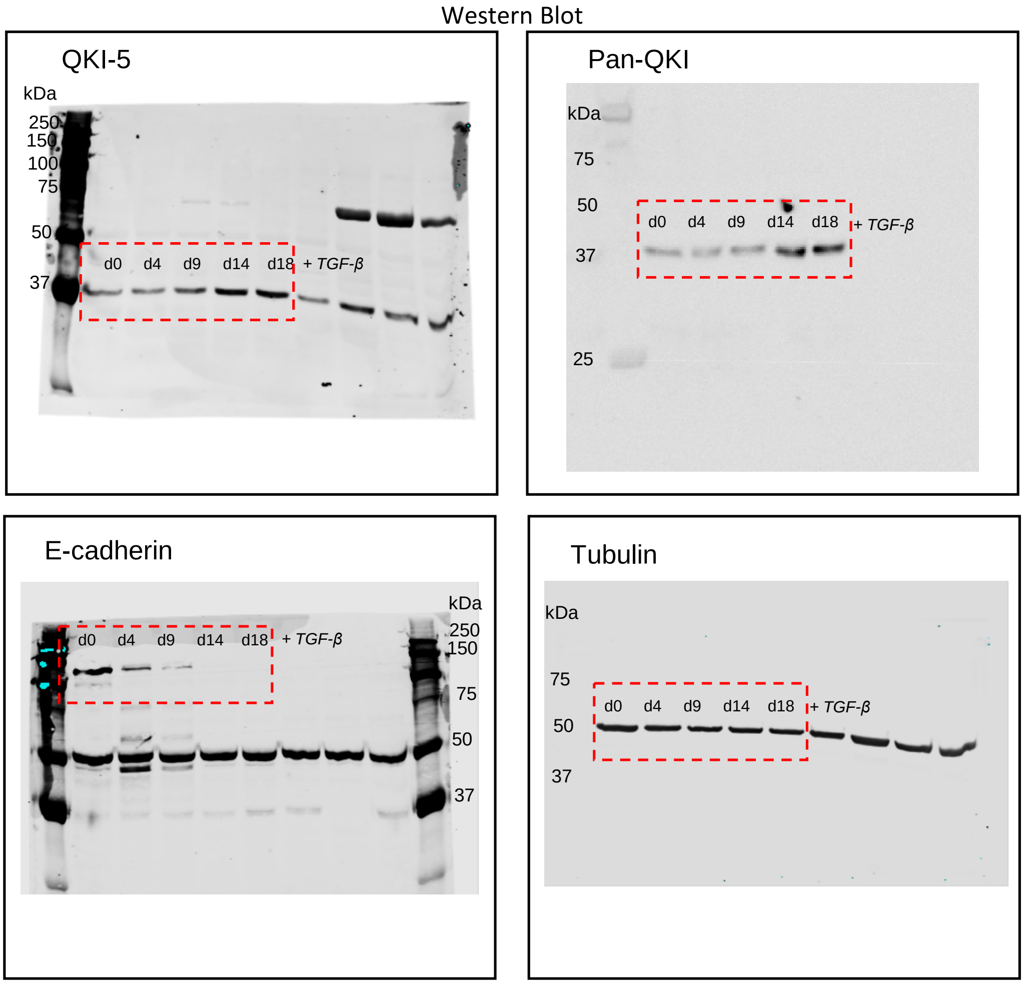

Figure 3 A - MCF10A

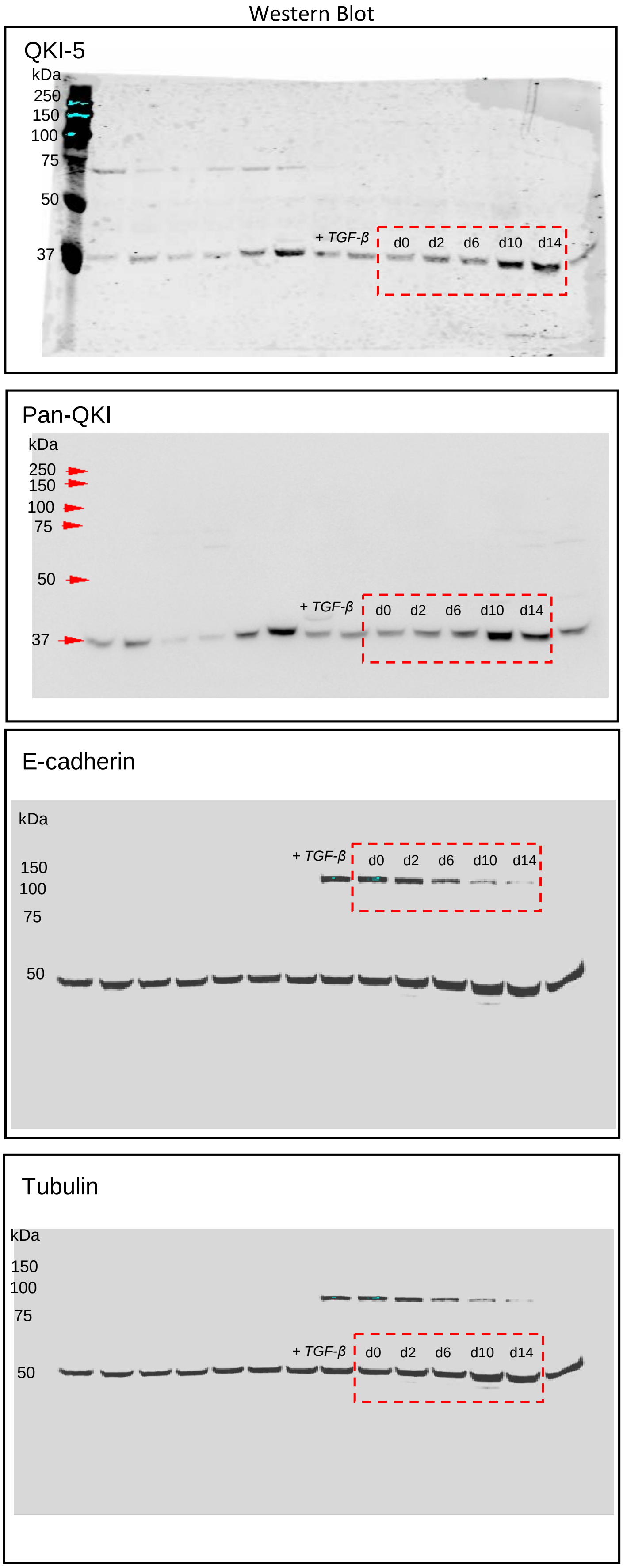

Figure 3 B - LNCaP-iZEB1

Western Blot

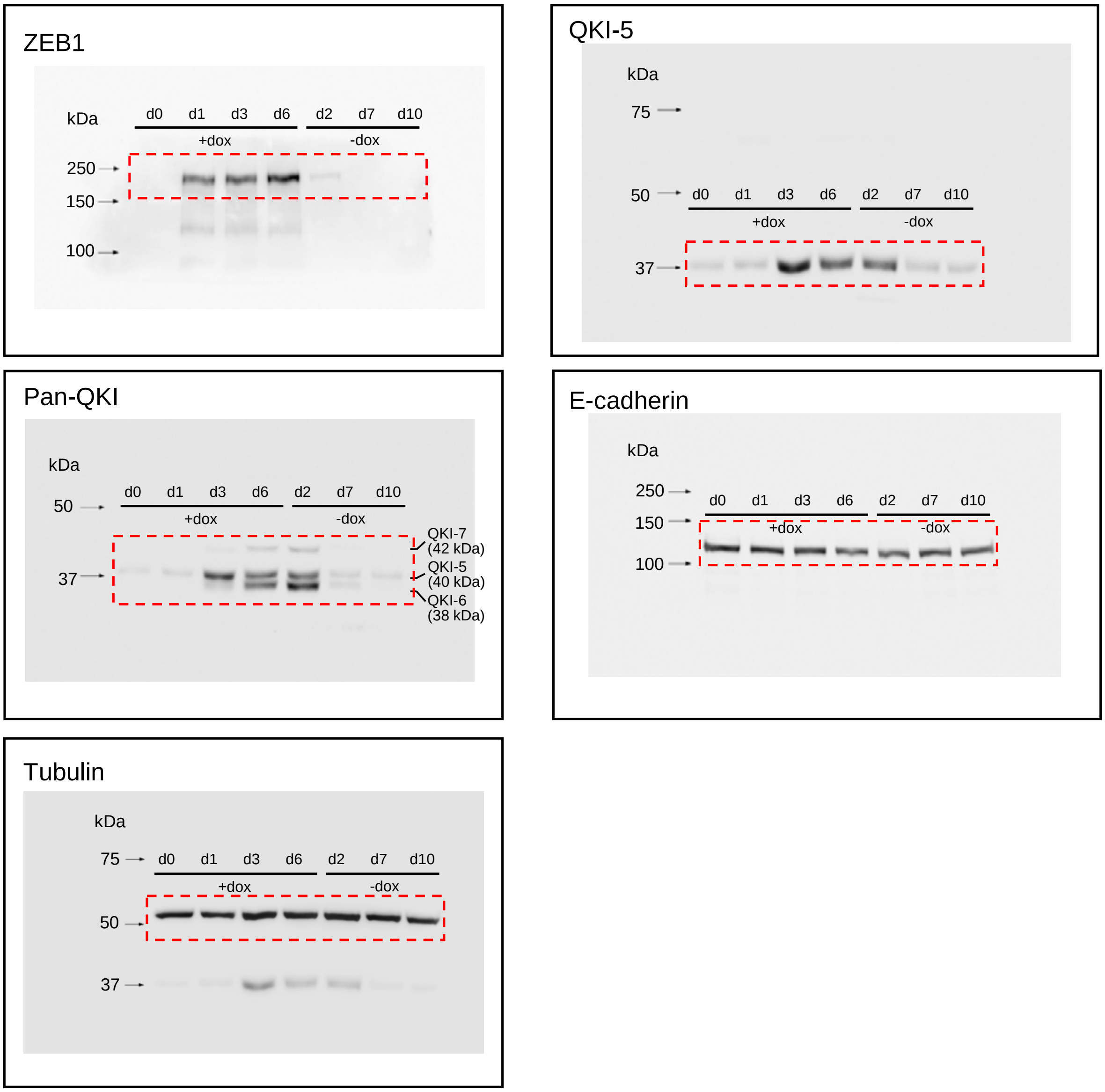

Figure 3 C - MCF7

Western Blot

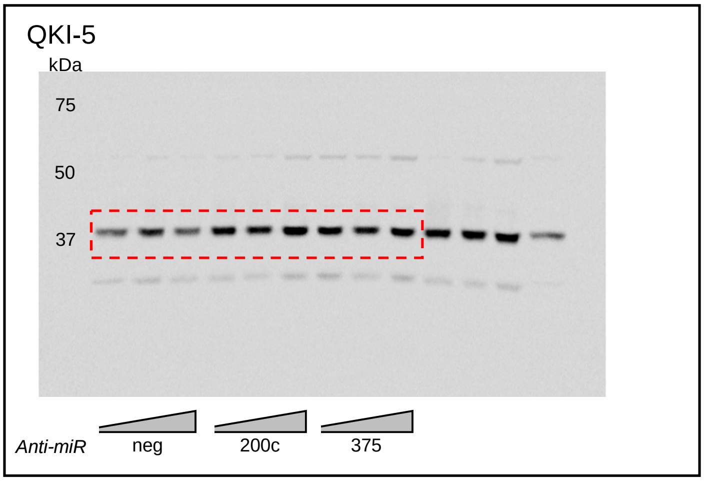


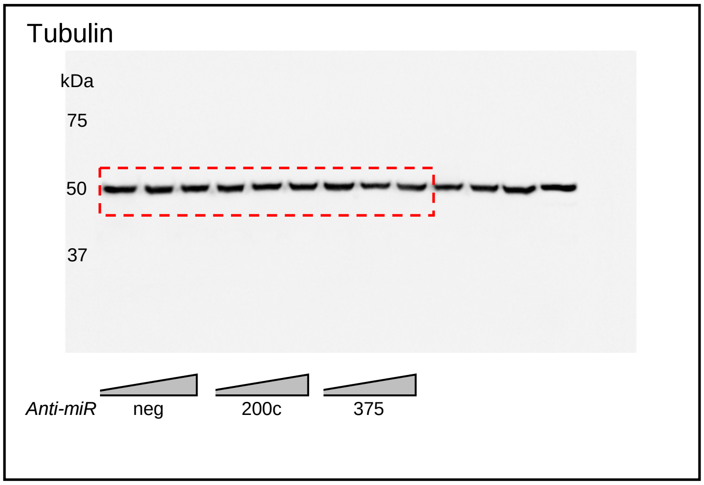

Supplement: Supplementary file 16 — Source Data for Figure 3 [file EMBJ-37-e99016-s014.pdf]
