# Supplementary material for: miR‐200/375 control epithelial plasticity‐associated alternative splicing by repressing the RNA‐binding protein Quaking
Source: EMBO J. 2018 Jun 6;37(13):e99016. doi: 10.15252/embj.201899016 (PMC6028027; doi:10.15252/embj.201899016)

Figure 4 C - SH-EP

Western Blot

QKI-5

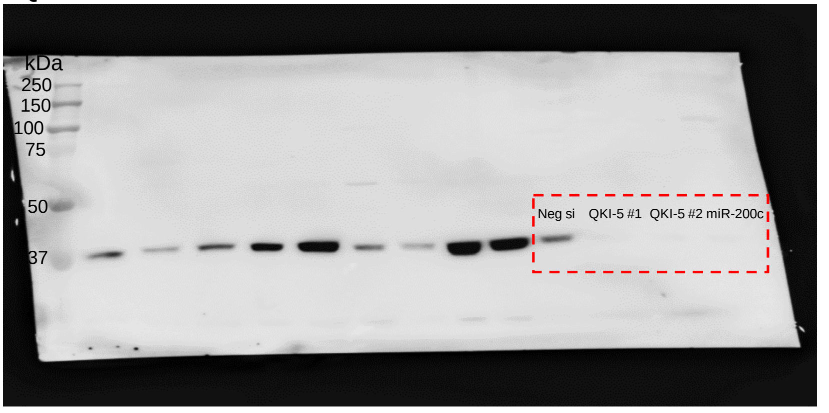

Tubulin

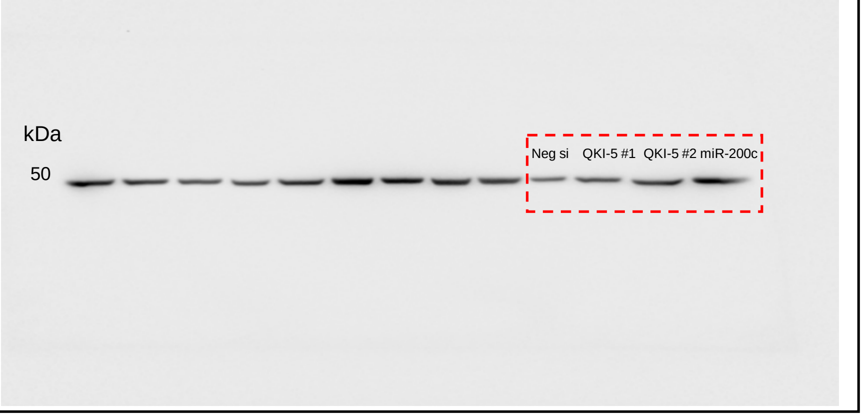

Figure 4 D - MDA-MB-231

Western Blot

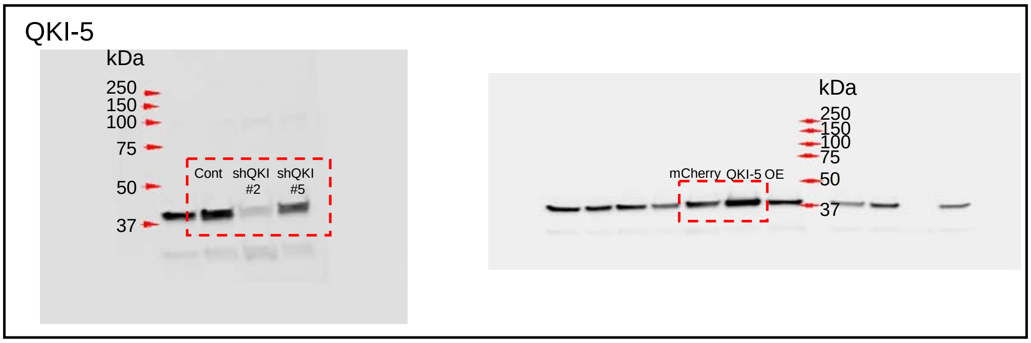

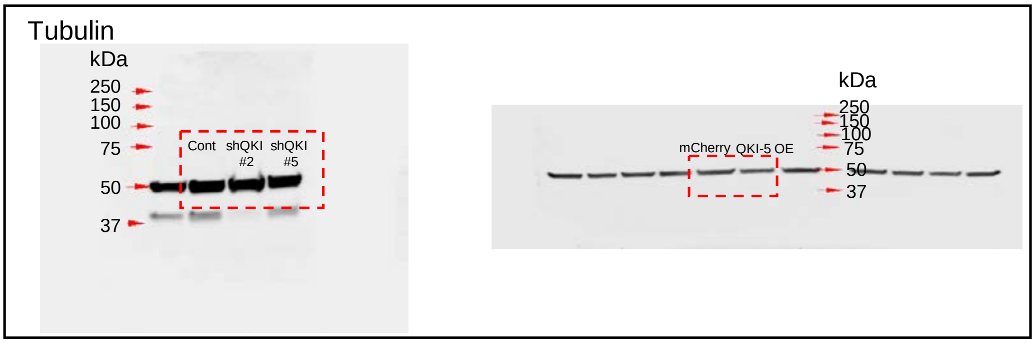

Figure 4 D - mesHMLE

Western Blot

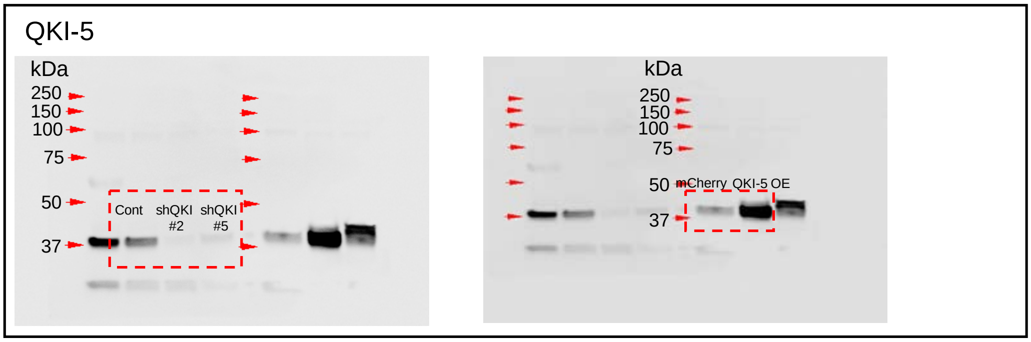

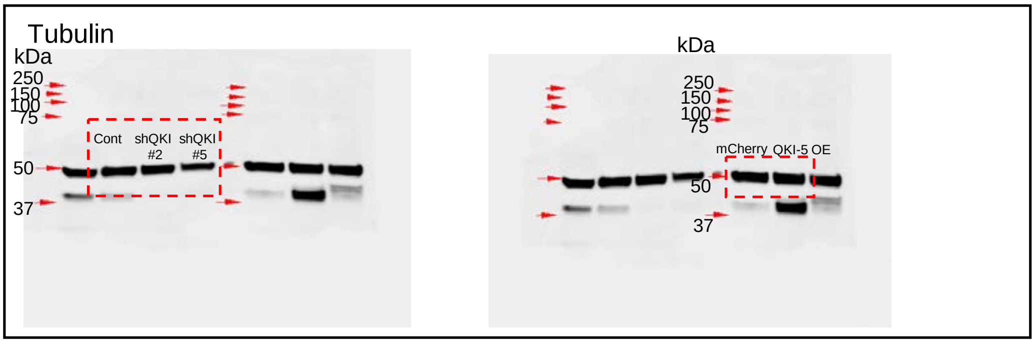

Figure 4 E – MDA-MB-231

Western Blot

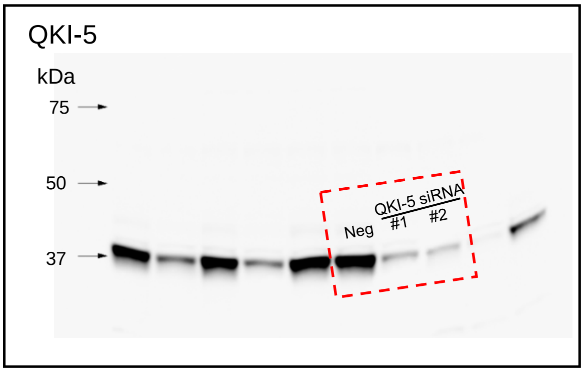

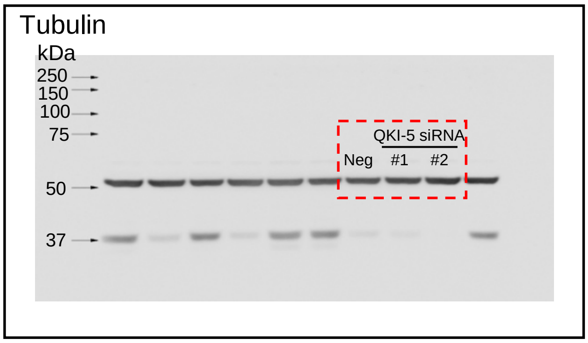

Figure 4 G – MDA-MB-231 LM2

Western Blot

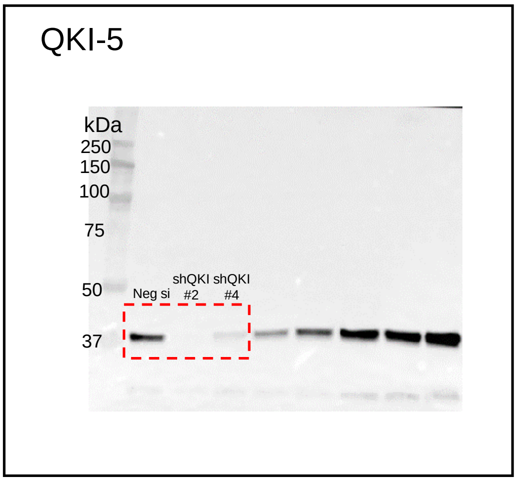

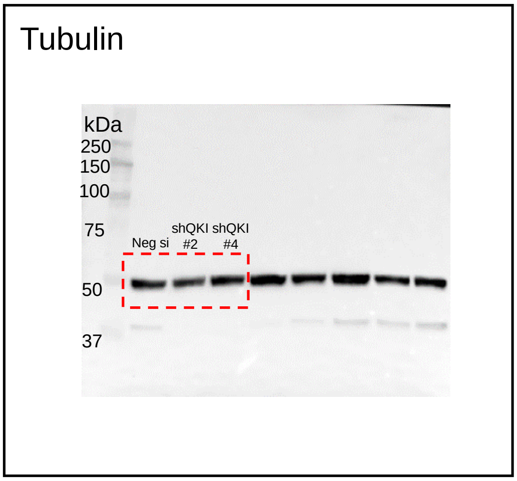

Supplement: Supplementary file 17 — Source Data for Figure 4 [file EMBJ-37-e99016-s015.pdf]
