# Supplementary material for: miR‐200/375 control epithelial plasticity‐associated alternative splicing by repressing the RNA‐binding protein Quaking
Source: EMBO J. 2018 Jun 6;37(13):e99016. doi: 10.15252/embj.201899016 (PMC6028027; doi:10.15252/embj.201899016)

Figure 5 F – HMLE/mesHMLE. Event for which QKI causes exon skipping

Splicing PCR

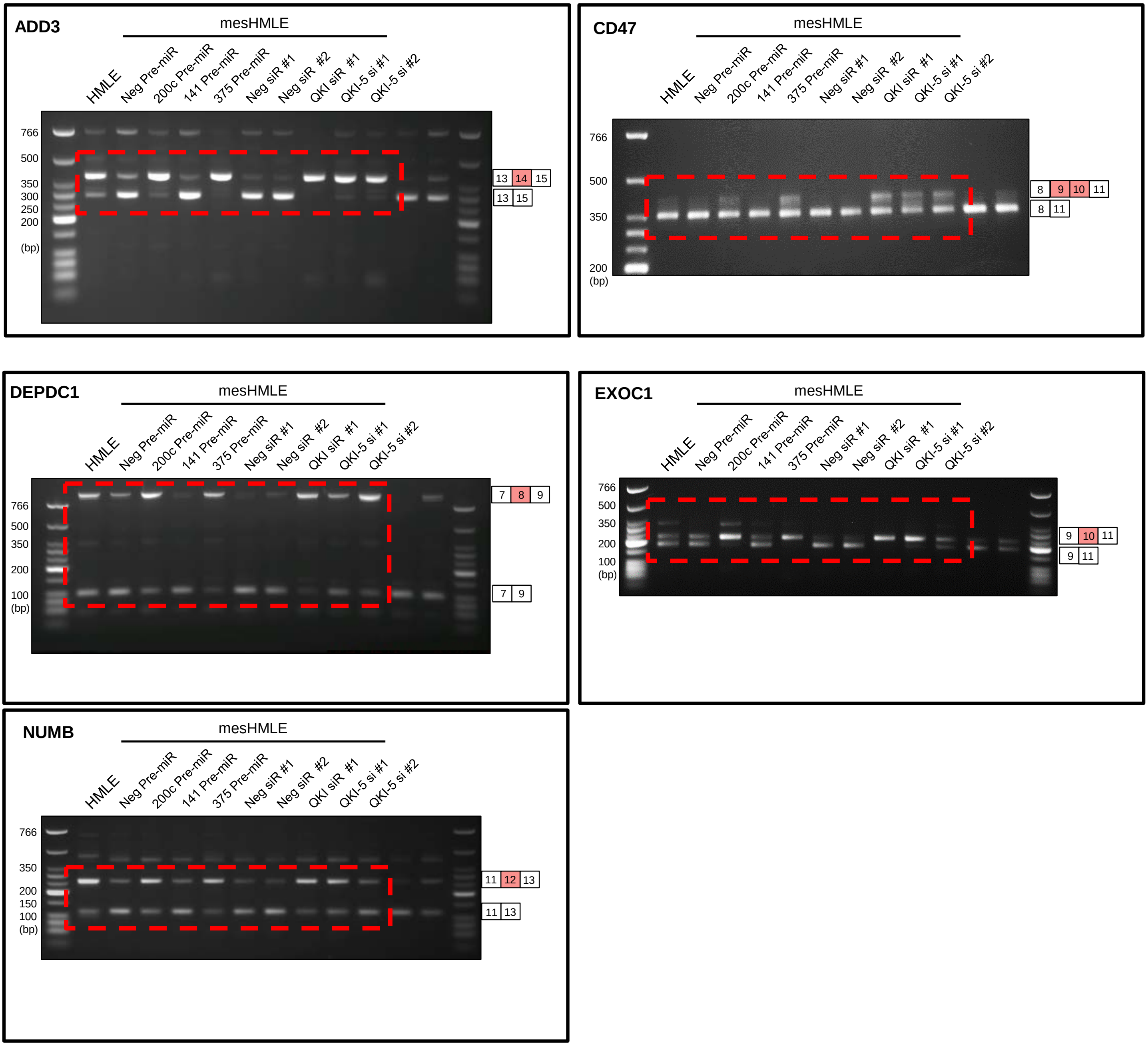

Figure 5 F – HMLE/mesHMLE. Event for which QKI causes exon inclusion:

Splicing PCR

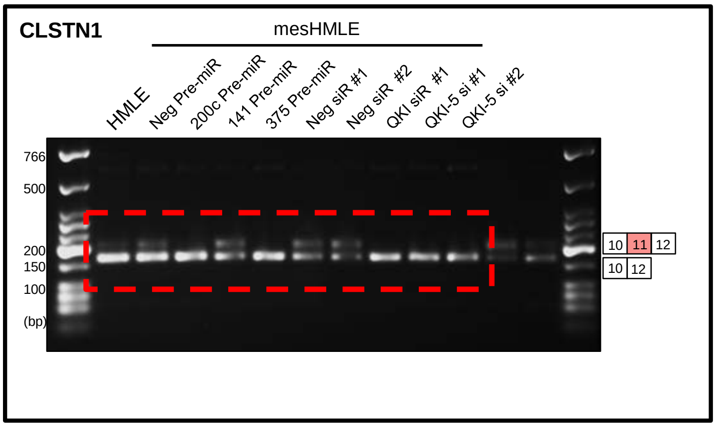

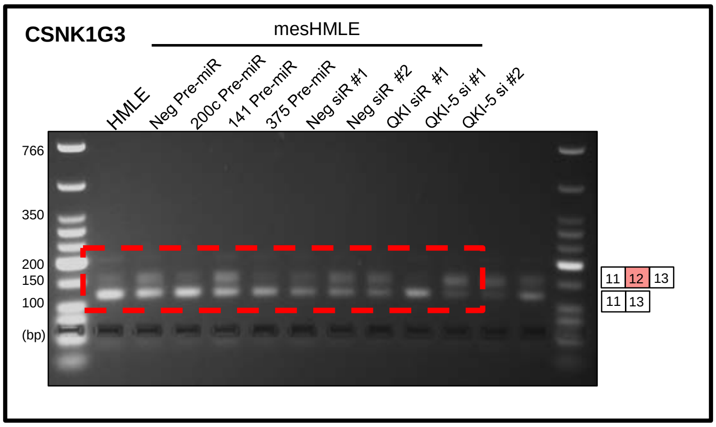

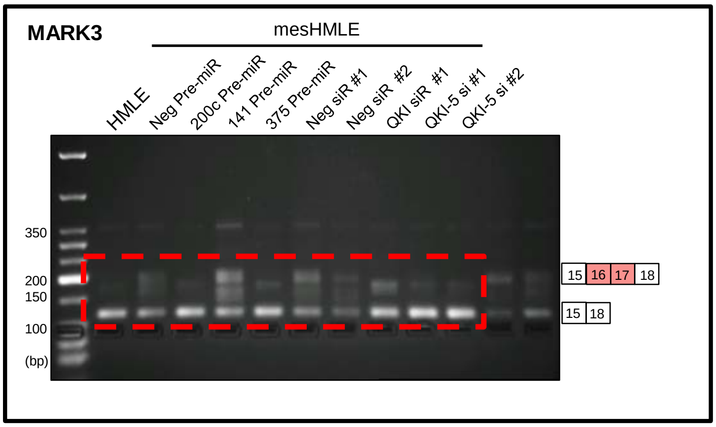

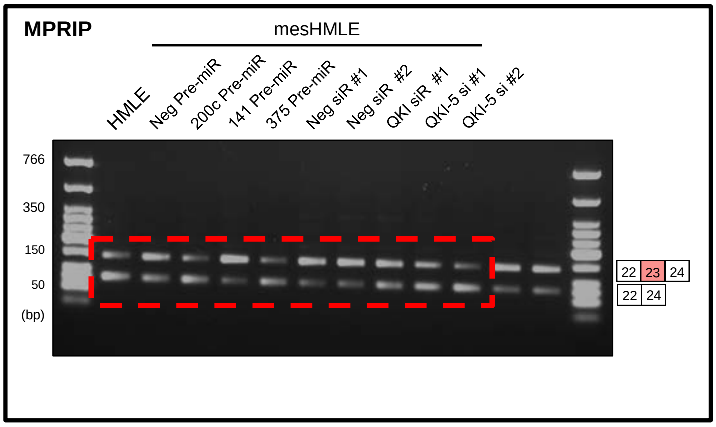

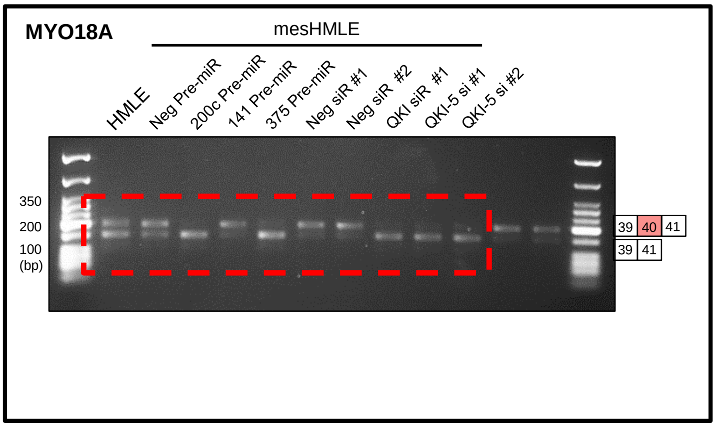

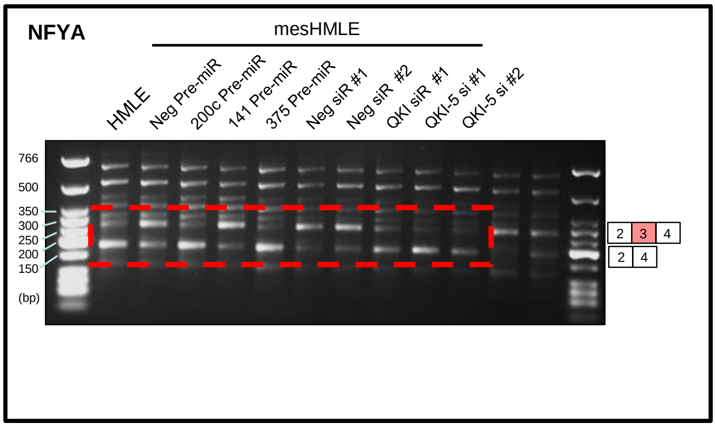

Figure 5 F – HMLE/mesHMLE. Normaliser

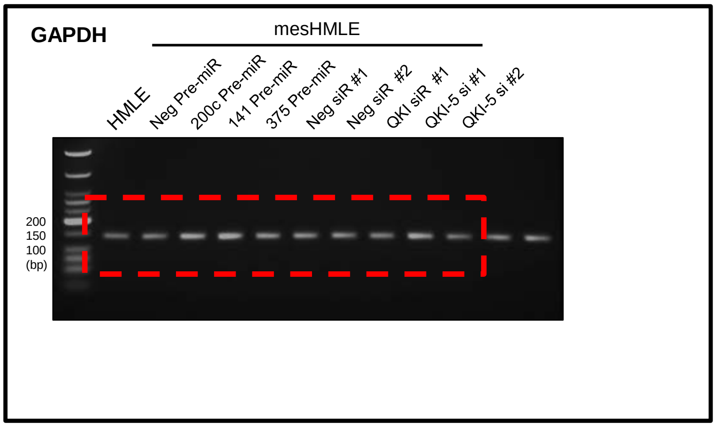

Figure 5 G – Panel of Breast Cancer Cell Lines. Events for which QKI causes exon skipping

Splicing PCR

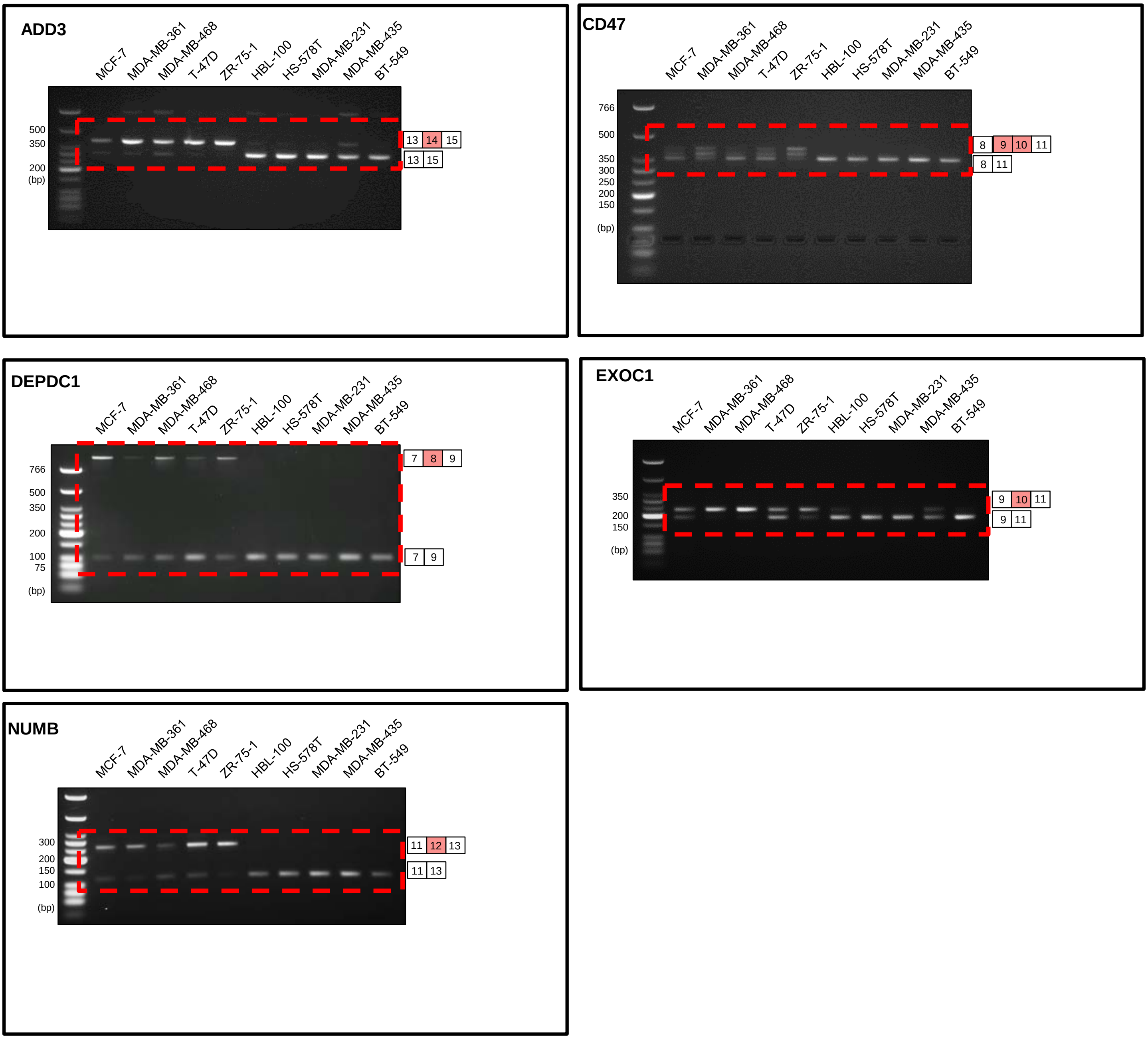

Figure 5 G – Panel of Breast Cancer Cell Lines. Events for which QKI causes exon inclusion

Splicing PCR

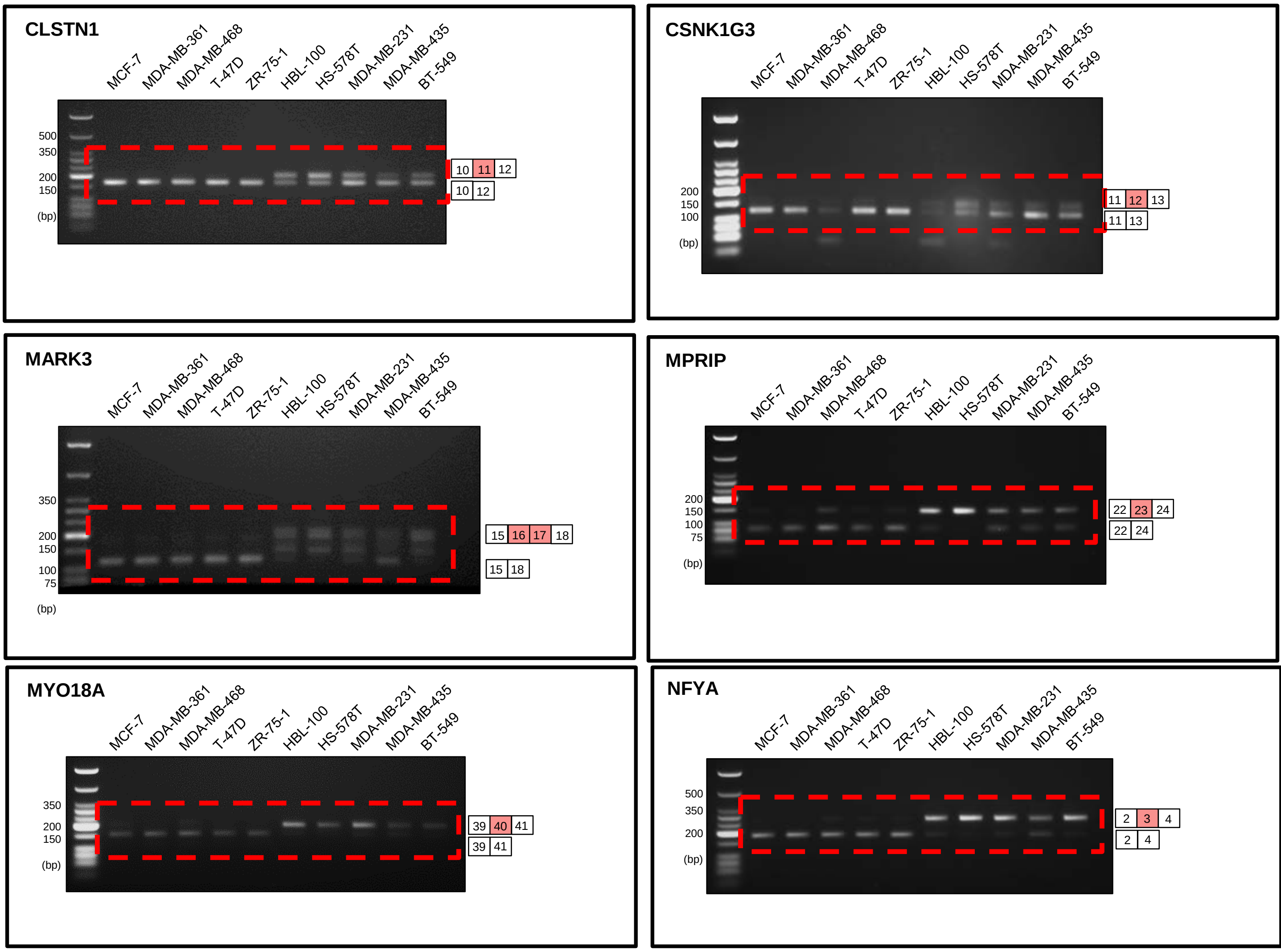

Figure 5 G – Panel of Breast Cancer Cell Lines. Normaliser

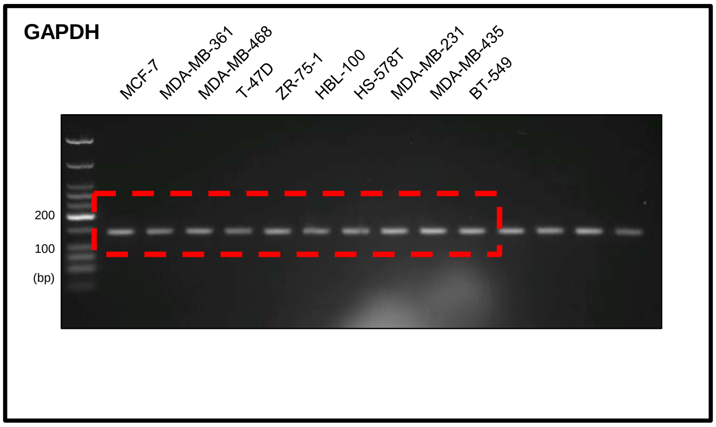

Supplement: Supplementary file 18 — Source Data for Figure 5 [file EMBJ-37-e99016-s016.pdf]
