# Supplementary material for: miR‐200/375 control epithelial plasticity‐associated alternative splicing by repressing the RNA‐binding protein Quaking
Source: EMBO J. 2018 Jun 6;37(13):e99016. doi: 10.15252/embj.201899016 (PMC6028027; doi:10.15252/embj.201899016)

Figure 6 A

Western Blot

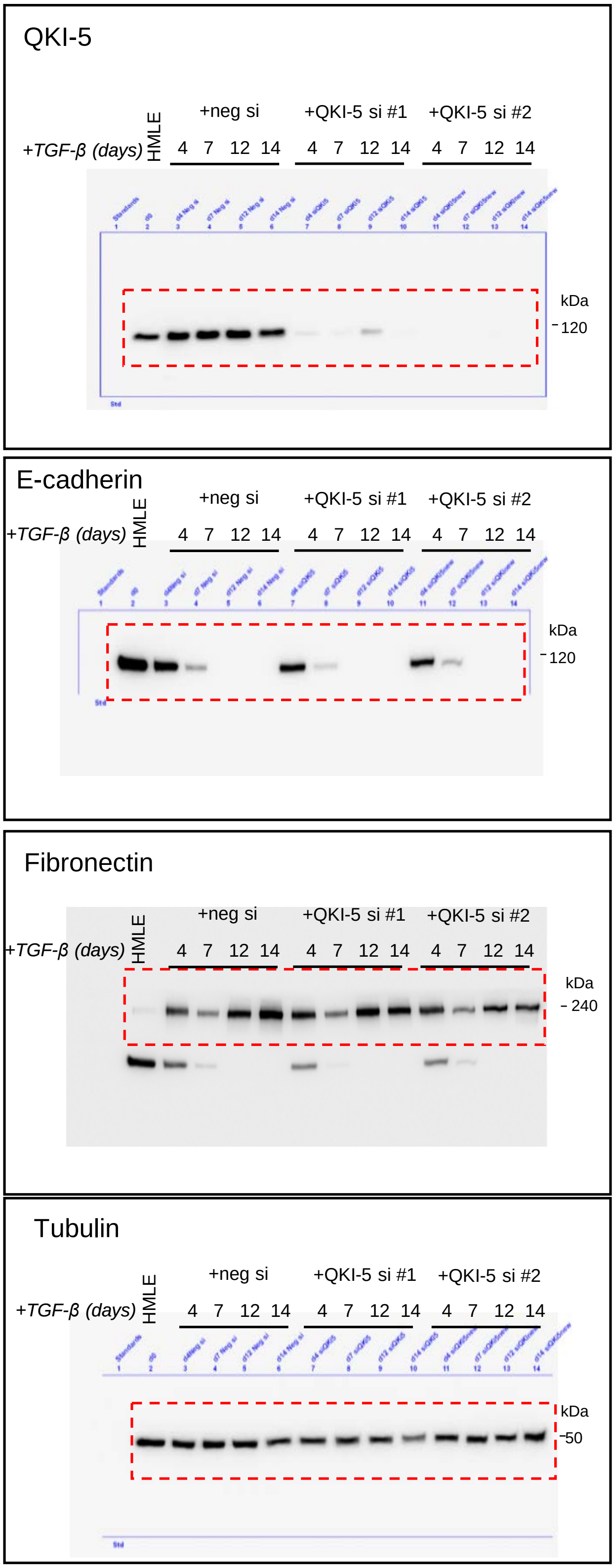

Splicing PCR

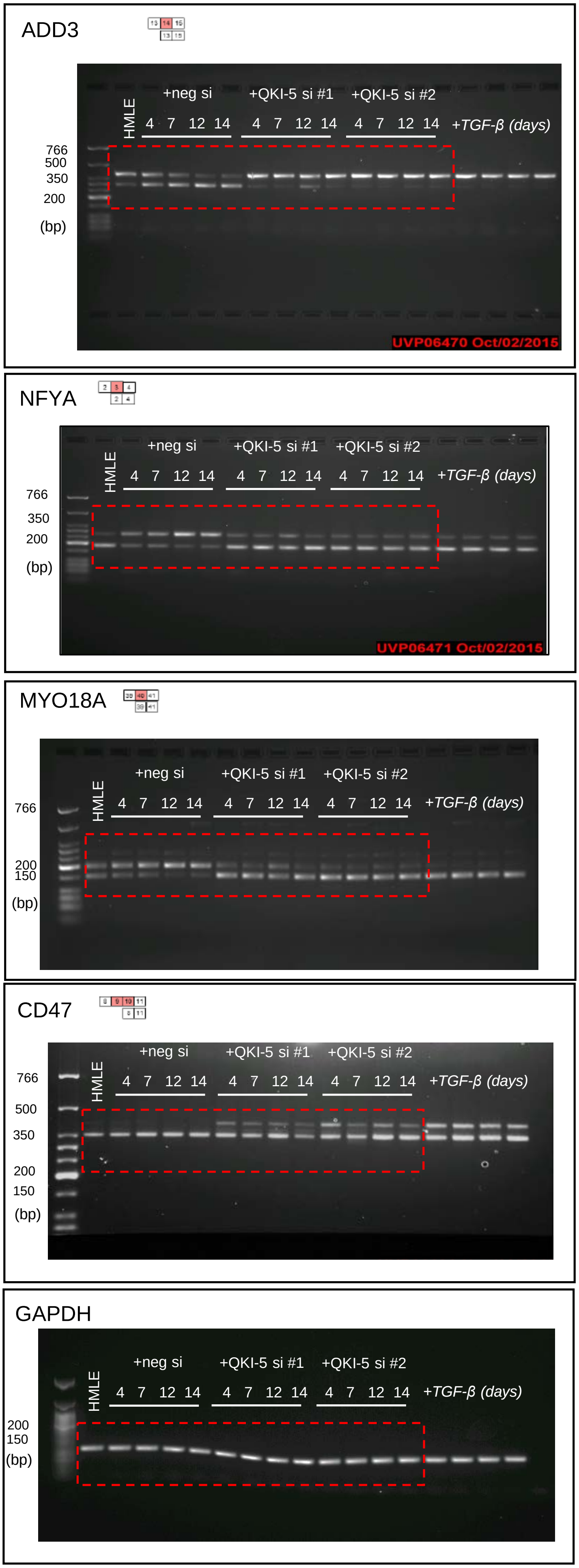

Figure 6 B

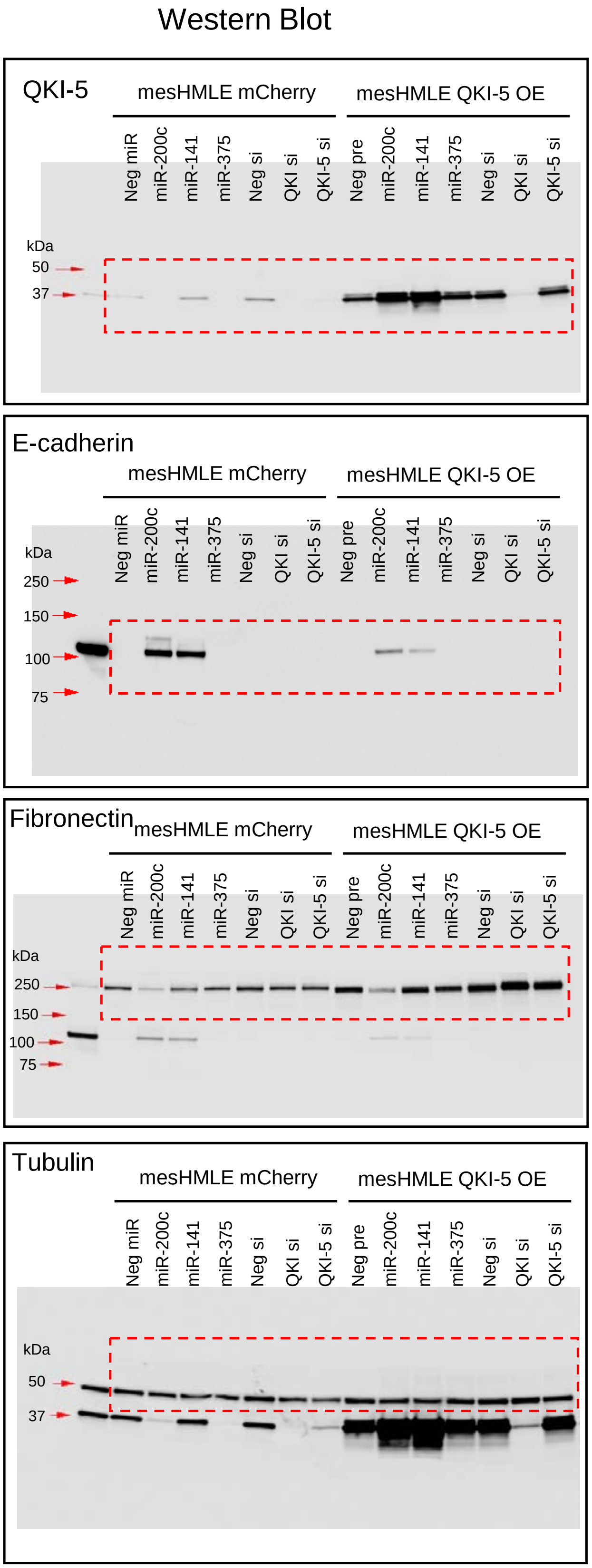

Western Blot
QKI-5
mesHMLE mCherry
mesHMLE QKI-5 OE
Neg miR
miR-200c
miR-141
miR-375
Neg si
QKI si
QKI-5 si
Neg pre
miR-200c
miR-141
miR-375
Neg si
QKI si
QKI-5 si
kDa
50
37
E-cadherin
kDa
250
150
100
75
Fibronectin
kDa
250
150
100
75
Tubulin
kDa
50
37

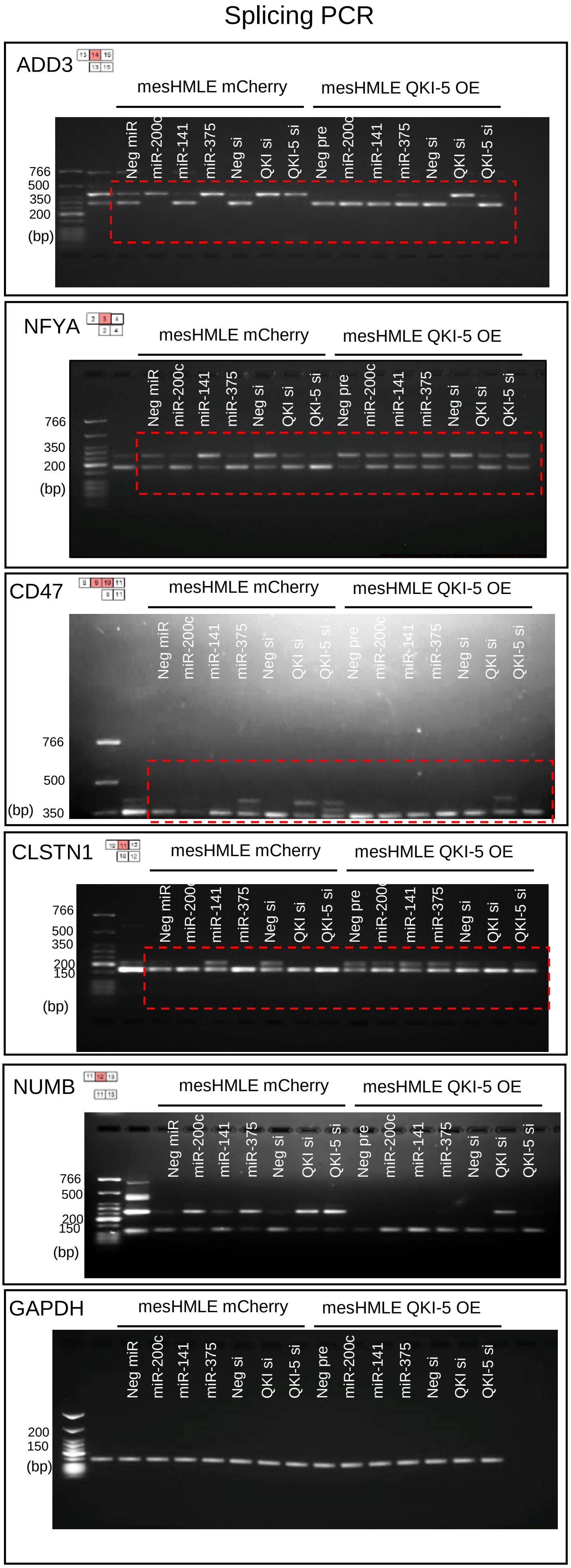

Splicing PCR
ADD3
mesHMLE mCherry
mesHMLE QKI-5 OE
Neg miR
miR-200c
miR-141
miR-375
Neg si
QKI si
QKI-5 si
Neg pre
miR-200c
miR-141
miR-375
Neg si
QKI si
QKI-5 si
766
500
350
200
(bp)
NFYA
766
350
200
(bp)
CD47
766
500
(bp)
350
CLSTN1
766
500
350
200
150
(bp)
NUMB
766
500
200
150
(bp)
GAPDH
200
150
(bp)

Figure 6 C

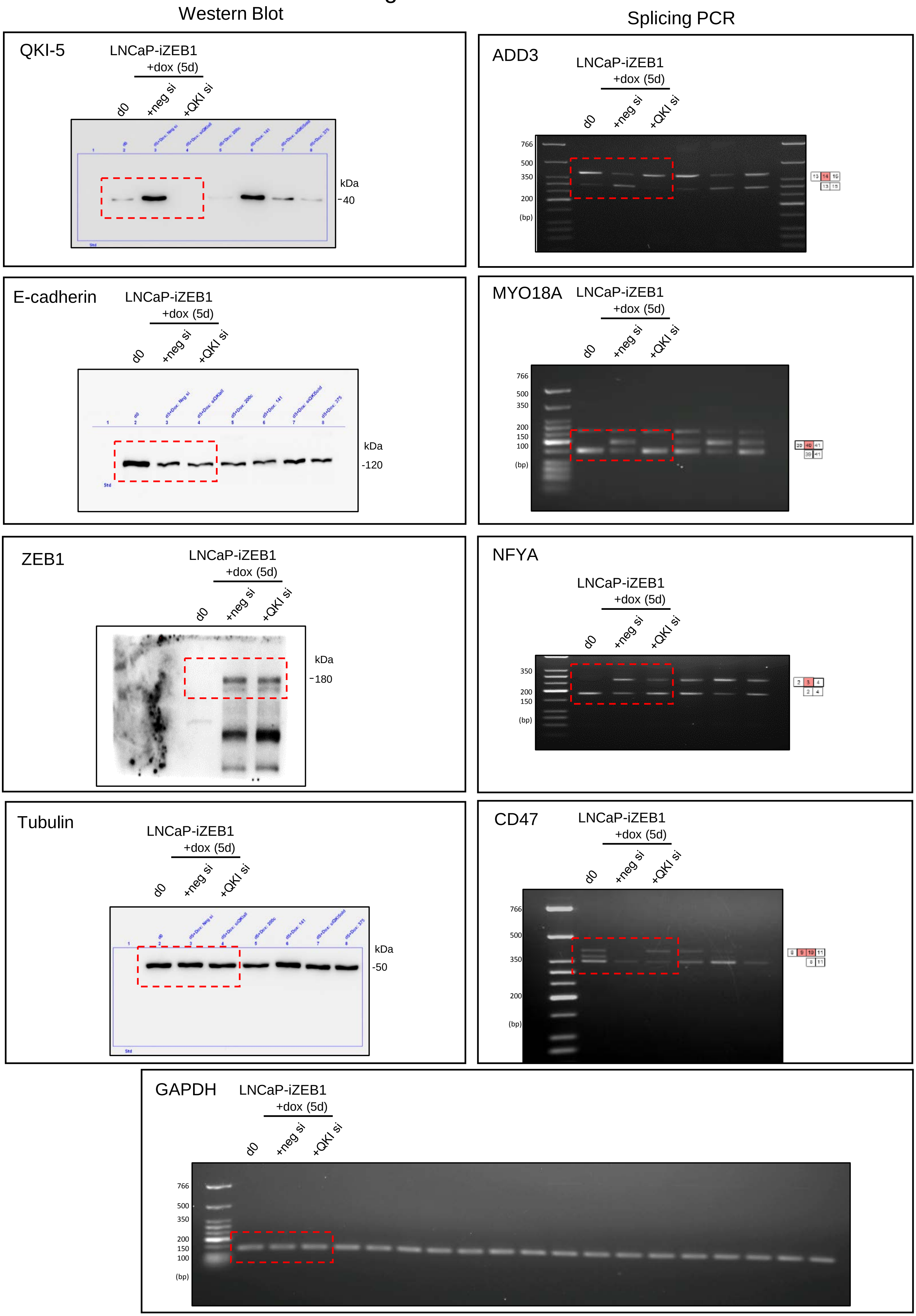

Western Blot
Splicing PCR
QKI-5
LNCaP-iZEB1
+dox (5d)
d0
+neg si
+QKI si
kDa
-40
ADD3
766
500
350
200
(bp)
E-cadherin
-120
MYO18A
150
100
ZEB1
-180
NFYA
Tubulin
-50
CD47
GAPDH

Figure 6 D

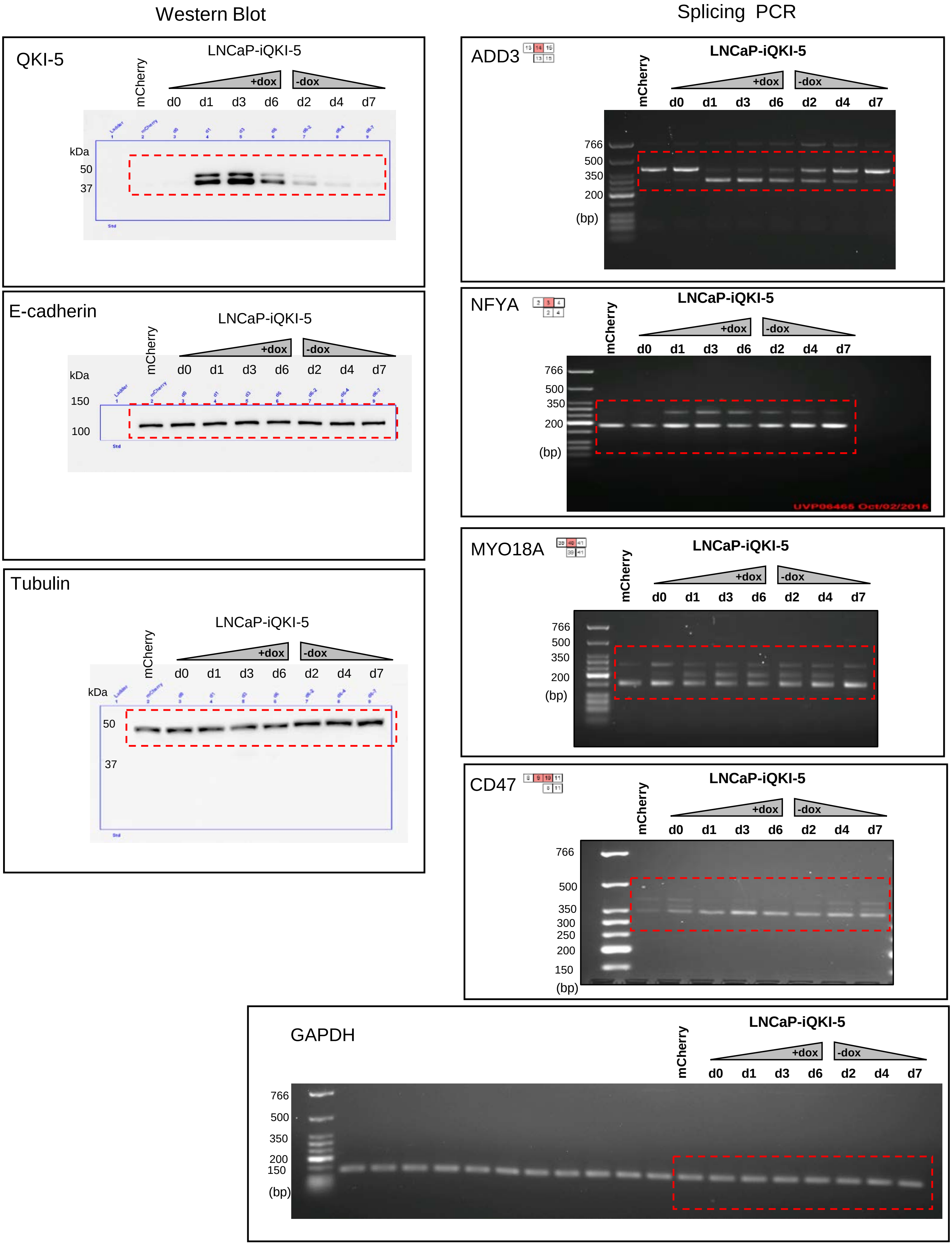

Western Blot
Splicing PCR
QKI-5
E-cadherin
Tubulin
LNCaP-iQKI-5
mCherry
+dox
-dox
d0 d1 d3 d6 d2 d4 d7
kDa
50
37
150
100
ADD3
NFYA
MYO18A
CD47
GAPDH
766
500
350
300
250
200
150
(bp)
UVP06465 Oct/02/2015

Figure 6 E

Western Blot

Splicing PCR

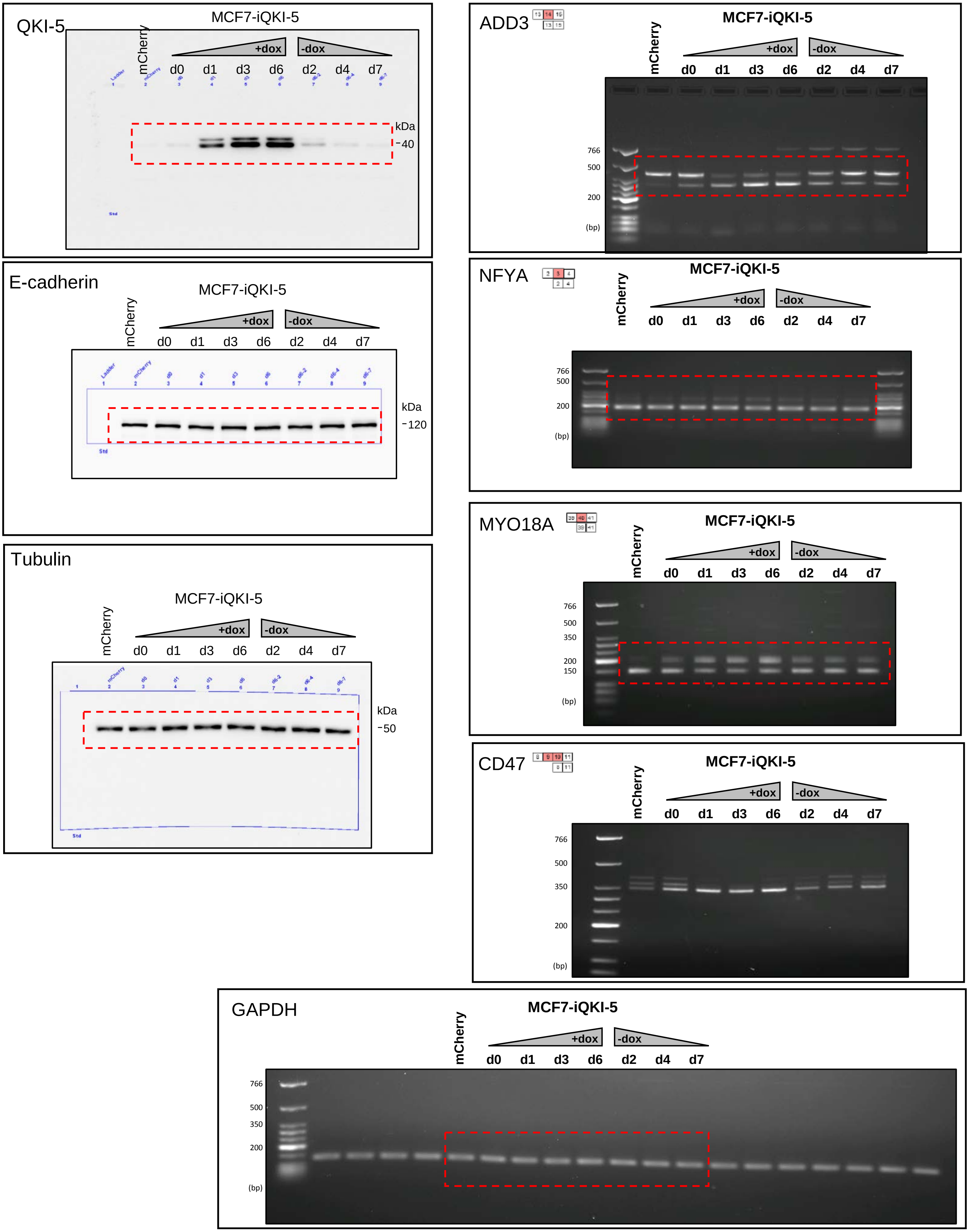

Supplement: Supplementary file 19 — Source Data for Figure 6 [file EMBJ-37-e99016-s017.pdf]
